# An Investigation of Stability and Species and Strain-Level Specificity in Bacterial Volatilomes

**DOI:** 10.3389/fmicb.2021.693075

**Published:** 2021-10-13

**Authors:** Shane Fitzgerald, Linda Holland, Aoife Morrin

**Affiliations:** ^1^School of Chemical Sciences, National Center for Sensor Research, SFI Insight Centre for Data Analytics, Dublin City University, Dublin, Ireland; ^2^School of Biotechnology, Dublin City University, Dublin, Ireland

**Keywords:** gas chromatography, microbial volatiles, headspace—solid phase microextraction, VOCs, MVOC (microbial volatile organic compounds), volatilomics, volatile organic compounds, wound infection—microbiology

## Abstract

Microbial volatilomics is a rapidly growing field of study and has shown great potential for applications in food, farming, and clinical sectors in the future. Due to the varying experimental methods and growth conditions employed in microbial volatilomic studies as well as strain-dependent volatilomic differences, there is limited knowledge regarding the stability of microbial volatilomes. Consequently, cross-study comparisons and validation of results and data can be challenging. In this study, we investigated the stability of the volatilomes of multiple strains of *Staphylococcus aureus, Pseudomonas aeruginosa* and *Escherichia coli* across three frequently used nutrient-rich growth media. Volatilomic stability was assessed based on media-, time- and strain-dependent variation across the examined bacterial volatilomes. Strain-level specificity of the observed volatilomes of *E. coli* and *P. aeruginosa* strains was further investigated by comparing the emission of selected compounds at varying stages of cell growth. Headspace solid phase microextraction (HS-SPME) sampling coupled with gas chromatography mass spectrometry (GC-MS) was used to analyze the volatilome of each strain. The whole volatilomes of the examined strains demonstrate a high degree of stability across the three examined growth media. At the compound-level, media dependent differences were observed particularly when comparing the volatilomes obtained in glucose-containing brain heart infusion (BHI) and tryptone soy broth (TSB) growth media with the volatilomes obtained in glucose-free Lysogeny broth (LB) media. These glucose-dependent volatilomic differences were primarily seen in the emission of primary metabolites such as alcohols, ketones, and acids. Strain-level differences in the emission of specific compounds in *E. coli* and *P. aeruginosa* samples were also observed across the media. These strain-level volatilomic differences were also observed across varying phases of growth of each strain, therefore confirming that these strains had varying core and accessory volatilomes. Our results demonstrate that, at the species-level, the examined bacteria have a core volatilome that exhibits a high-degree of stability across frequently-used growth media. Media-dependent differences in microbial volatilomes offer valuable insights into identifying the cellular origin of individual metabolites. The observed differences in the core and accessory volatilomes of the examined strains illustrate the complexity of microbial volatilomics as a study while also highlighting the need for more strain-level investigations to ultimately elucidate the whole volatilomic capabilities of microbial species in the future.

## Introduction

For many years the occurrence of disease-specific volatiles have been used as a supporting factor in the clinical diagnoses of various disorders, e.g., the sulfide emission in the breath of *Helicobacter pylori*-positive patients with gastrointestinal issues (Hoshi et al., 2002); and the sweaty feet odor of patients with isovaleric acidemia (Budd et al., 1967). As a result, the study of volatile organic compounds (VOCs) produced by commensal and pathogenic microorganisms has emerged as a path to characterizing these disease-specific volatiles. In particular, the last 15 years has seen a significant rise in the study of microbial VOCs due to the universal implementation of improved analytical methodology and data analysis techniques. Comprehensive sampling and analytical methods have broadened the spectrum of compounds that can be investigated while the incorporation of dimension reduction and clustering methods has enabled the identification of discriminatory trends across the microbial VOC data. Most studies have been primarily focused on the investigation of *in vitro* microbial cultures and have been critical in identifying metabolic and cellular pathways of particular compounds. These studies have demonstrated that species-level differences in VOC production do exist between pathogenic and commensal microbial species and highlight the need for further study.

The diversity and mechanisms behind microbial volatilomes have been recently illustrated in several comprehensive review papers (Schulz and Dickschat, 2007; Elmassry and Piechulla, 2020; Kai, 2020; Weisskopf et al., 2021) and books (Ryu et al., 2020). Growth parameters such as growth media (Jenkins and Bean, 2019, 2020), growth phase (Filipiak et al., 2012b; Chen et al., 2016), oxygen content (Insam and Seewald, 2010), temperature (Misztal et al., 2018) and pH (Schulz-Bohm et al., 2017), all influence microbial volatilomes. Another less studied factor in the overall variation seen across the volatilomes of microbial species is the occurrence of strain-level specificity in volatilomic emission within a given species (Schulz et al., 2020; Weisskopf et al., 2021). In addition to this, the variation in sampling [SPME (Timm et al., 2018; Fitzgerald et al., 2020), thermal desorption tubes (Filipiak et al., 2012a,b), direct syringe (Ashrafi et al., 2018a,b)] and analytical techniques [gas chromatography–mass spectrometry (GC-MS) (Filipiak et al., 2012b; Timm et al., 2018; Fitzgerald et al., 2020), selected ion-flow-tube (SIFT) MS (Shestivska et al., 2015), proton transfer reaction (PTR) MS (Bunge et al., 2008)] employed across the field also have a direct influence on the VOC profiles reported in the literature. Consequently, cross-study validation of reported microbial VOC profiles remains a major challenge in the field. However, the recent establishment of the mVOC 2.0 database (Lemfack et al., 2017) has allowed for some qualitative comparisons of microbial volatilomes and will evolve to be a valuable tool in the field. The database contains thousands of logged compounds along with growth conditions and analytical methods used to acquire the volatilomes of a wide range of microbes. With such a platform available, a community-wide effort is required to build on it and to ensure that the microbial volatilomic profiles available on the database are as comprehensive as possible with respect to the literature. This will ultimately allow the full examination of individual microbial volatilomic profiles relative to all the conditions in which they have been previously examined. Therefore, in order to elucidate the full spectrum of microbial volatilomes, there is a strong need to investigate them under varying conditions in controlled settings.

*Staphylococcus aureus, P. aeruginosa*, and *E. coli* are highly prevalent wound pathogens and are responsible for particularly severe infections in diabetic foot ulcers (DFUs) (Gardner et al., 2012; Ndosi et al., 2017). It is estimated that around one in four people with diabetes will develop a diabetic foot ulcer (DFU) in their lifetime (Armstrong et al., 2017). Infections of DFUs highly increase the risk of poor outcomes such as amputation (Abdulrazak et al., 2005). The duration of the DFU is proportional to its severity and is closely associated with species- and strain-level microbial diversity within the infection (Abdulrazak et al., 2005; Kalan et al., 2019). Currently in clinics, time consuming techniques such as blood tests and traditional plate-based techniques are employed to detect potential infections (Lipsky et al., 2019). As early detection is critical in preventing severe infections, rapid non-invasive detection of pathogenic bacterial volatiles in wounds and wound samples could potentially speed up the turnover of clinical information and greatly contribute to the clinical workflow. Our group is currently working on detecting pathogen-specific volatile compounds in DFU swab samples. Volatilomic profiling of pure microbial cultures has played a critical role in our preparation for clinical volatilomic work and also for the interpretation of the data obtained.

The stability of bacterial volatilomes both in different nutritional conditions and strain-to-strain remains relatively understudied. In this study, we examined multiple strains of *E. coli, P. aeruginosa*, and *S. aureus* volatilomes across different growth media. By examining microbial volatilomic variability across different strains and different media, the core and accessory volatilomes of these bacteria can be elucidated. These terms were introduced by Bean et al. (2016) but for the context of this study, core compounds refer to compounds that are emitted by both strains across all media; accessory compounds are compounds emitted by at least one strain in at least one medium. Our key objectives of this work were (1) to obtain comprehensive volatilomic data for multiple strains of *S. aureus, P. aeruginosa*, and *E. coli* in three different growth media (BHI, LB, TSB); (2) to assess the stability and variation of the observed bacterial volatilomes; and (3) to temporally investigate strain-level specificity within the selected volatilomes by comparing the emission of specific compounds at progressive stages of growth and development of the cells.

## Materials and Methods

### Growth of Bacteria

The following bacterial strains were examined: *S. aureus* (DSM2569 and DSM799)*; P. aeruginosa* (DSM19880 and DSM25642)*; E. coli* (DSM30083 and DSM105372). All *S. aureus, P. aeruginosa*, and *E. coli* isolates were obtained from Leibniz Institute DSMZ-German Collection of Microorganisms and Cell Cultures GmbH. Each strain was streaked individually on tryptone soy broth (TSB) agar media plates. For each replicate, a single colony was inoculated in 4 mL of TSB, BHI, or LB broth and incubated at 37°C overnight. Each replicate overnight culture was individually incubated in a 50 mL conical centrifuge tube. Each overnight culture was diluted to a total volume of 5 mL in growth media (BHI, TSB, LB, NB) to a cell count of approximately 10^8^–10^9^ colony forming units (CFU)/mL in the 20 mL headspace vials which were then sealed with magnetic Polytetrafluoroethylene/silicone septum screw caps (Merck, Cork, Ireland). For each of the examined growth media, five samples of each strain were incubated at 37°C and shaking for 24 h—after which point the headspace (HS) of each sample was directly sampled and analyzed (described below).

The samples are referred throughout the text using the following acronyms*: EC.A: E. coli DSM103372, EC.B: E. coli DSM30083, PA.A: P. aeruginosa DSM105372, PA.B: P. aeruginosa DSM25642, SA.A: S. aureus DSM2569, SA.B: S. aureus DSM799, TSB: Tryptone soy broth (OXOID: CM0129), BHI: Brain heart infusion (OXOID: CM1135), LB: Lysogeny broth (SIGMA: L3022; NaCl 5 g/L), and NB: Nutrient broth (OXOID: CM0001).*

### Growth Curve Analysis

Growth curve analysis was performed on *P. aeruginosa* and *E. coli* samples (*n* = 3). Bacterial samples were diluted to an initial OD_600_ of 0.1 which corresponded to a cell count of 10^8^–10^9^ cfu/mL. Prior to each round of solid phase microextraction (SPME) sampling, the OD_600_ of each sample was measured by extracting 20 μL from the culture using a stainless steel needle and syringe. This was done by piercing the needle through the septum of the HS vial and tilting the vial to extract the small volume of culture. OD_600_ was measured at 1, 2, 3, 4, 5, 6, 7, 8, and 24 h.

### Volatile Organic Compound Sampling Procedure

Solid phase microextraction fibers were used for sampling VOCs and consisted of 85 μm Carboxen/Polydimethylsiloxane Stableflex (2 cm) assemblies (Supelco Corp., Bellefonte, PA, United States). Prior to sampling, each bacterial or control sample was removed from the shaking incubator and placed in a standard incubator at 37°C. The SPME needle was pierced through the septum of the HS vial, and the fiber was exposed to the HS of the sample for 20 min while agitated. Following this, the fiber was retracted and the SPME assembly removed from the vial. The SPME fiber was then inserted into the GC inlet and thermally desorbed at 250°C for 2 min for subsequent separation and detection by mass spectrometry. During the temporal analysis, the magnetic screw caps of each sample were also tightly covered with parafilm following each round of sampling to minimize any loss of VOCs.

### Gas Chromatography–Mass Spectrometry

An Agilent 6890 GC connected to an Agilent 5973 mass selective detector (Agilent Technologies, Inc., Santa Clara, CA, United States) was used for all analyses. Separations were performed on a DB-WAX column (Agilent Technologies Ireland, Cork) (30 m × 0.25 mm × 0.32 μm). The carrier gas used was helium, with a constant flow rate of 1.3 mL/min For manual injections of SPME fibers, the system was equipped with a SPME Merlin Microseal (Merlin Instrument Company, Newark, DE, United States), and the inlet was maintained at a temperature of 250°C. Split-less injection was used for all samples, with a gas purge being activated after 2 min. Each SPME fiber was desorbed for 2 min within a SPME inlet liner (Supelco). The initial GC oven temperature was 40°C for 5 min and was programmed to increase at a rate of 10°C min^–1^ to 240°C, with a final hold for 5 min at this temperature, giving an overall running time of 29 min. The transfer line temperature was set at 230°C. The MS was operated at a scan range of 35–400 *m/z*, scan rate of 3.94 s^–1^, ion source temperature 230°C and ionizing energy of 70 eV. Identification of compounds was performed using the National Institute of Standards and Technology (NIST) library (2017)—match factors of >70% were used. Retention index (RI) values for polar columns provided by the NIST Chemistry WebBook, SRD 69, was used to support the identification of these compounds. Any compound found to have an RI value ≤ 12 RI units of the RI values found in the NIST database were deemed acceptable matches. An external standard mixture of saturated alkanes (C_7_–C_30_; Merck, Cork, Ireland) was injected into the GC-MS under the same temperature conditions as the samples and used for RI matching. This was done by rapidly dipping an exhausted SPME needle into the mixture once and injecting it into the GC-MS. A fully functional SPME fiber was not used for this because exposure to hexane degrades the fiber integrity.

### Data Analysis

Agilent MassHunter Qualitative Analysis 10.0 software was used to analyze raw chromatographic data. Peak acquisition and the respective peak area data were calculated by employing the chromatogram deconvolution compound mining algorithm. Chromatographic peaks were compared using the NIST Chemistry WebBook. Peaks found to be from exogenous sources such as the SPME fiber, glass vial, and column were removed from the dataset. Only peaks that could be accurately identified and that were detected in over one replicate sample were included in the final peak list. R (version 1.2.5033) was used for data exploration and visualization. Raw bacterial VOC data was standardized using centering and scaling (van den Berg et al., 2006). Centering converts all the values in the dataset to fluctuations around zero rather than fluctuations around the mean VOC abundance. It adjusts for differences in the offset between low and high abundances. Scaling converts the values in the dataset into ratios relative to the difference in abundances between the VOCs, which allows each VOC to be equally represented in the subsequent data analysis. For compounds that were present in some replicate samples (of a given strain in a given media) and absent from others, these missing values were inputted as zero. For compounds that were absent from all replicates (of a given strain in a given media), these missing values remained missing values. Hierarchical clustering and principal component analysis (PCA) were carried out on the dataset using the R packages: “FactoMineR” (version: 2.4), “factoextra” (version: 1.0.7), “pheatmap” (version: 1.0.12), “egg” (version: 0.4.5) and “cluster” (version:2.1.0). For the hierarchical clustering analysis, Euclidean distance was used as the measure of (dis)similarity. Other R packages used for the graphics in this study were: “tidyverse” (version: 1.3.1), “ggplot2” (version: 3.3.5), “ggfortify” (version: 0.4.12).

## Results

### Stability of Core Volatile Organic Compound Profile Across Nutrient-Rich Media

**FIGURE 1:**
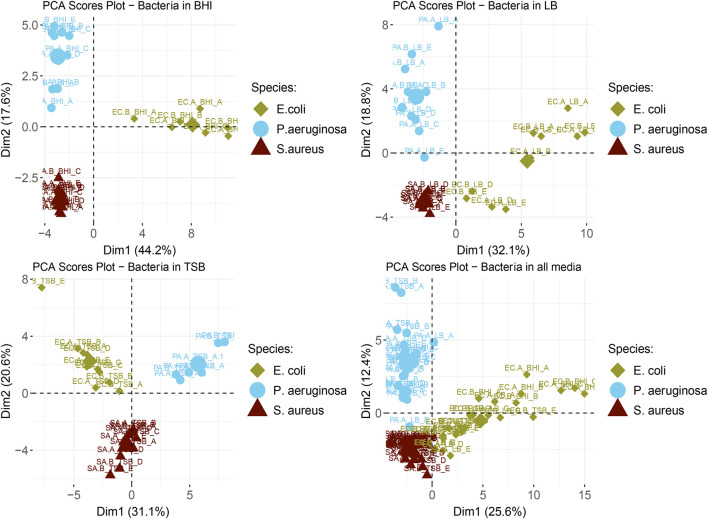
Labeled scores plot representations of scores from the PCA analyses of bacterial samples in BHI (top left), LB (top right), TSB (bottom left), and all examined media (bottom right). The large symbols in each plot are the geometric means for each species. The corresponding strain names to the abbreviated titles of the bacterial samples shown in this plot are as follows: EC.A: *E. coli* DSM103372, EC.B: *E. coli* DSM30083, PA.A: *P. aeruginosa* DSM105372, PA.B: *P. aeruginosa* DSM25642, SA.A: *S. aureus* DSM2569, SA.B: *S. aureus* DSM799.

The PCA scores plots shown in Figure 1 and the heatmaps shown in Supplementary Figures 1–3 visualize the similarities and dissimilarities between the bacterial samples across three different nutrient-rich growth media. Across the three examined growth media, a total of 64 compounds were used to investigate the overall discrimination of observed VOC profiles at the species-level. For the unsupervised analyses, whole bacterial volatilomes were analyzed based on 55 compounds in BHI (Figure 1, top left); 57 compounds in LB (Figure 1, top right); and 49 compounds in TSB (Figure 1, bottom left). In Figure 1, each sample is color coded based on its respective species. Similar to our previous results (Fitzgerald et al., 2020), the variation between *S. aureus* and *P. aeruginosa* samples was summarized by PC2 (y-axis), while the variation between *E. coli* and the other bacteria was summarized by PC1 (x-axis). *S. aureus* samples appear to have the most stable volatilome across the three media as they are tightly clustered together in the bottom left corner of the plot. *P. aeruginosa* has a slightly higher degree of media-dependent distribution of samples as it can be seen that the VOC profiles of samples cultured in TSB appear to be less variable than that of samples cultured in BHI and LB. In contrast to this, in *E. coli* samples, a relatively high degree of variability was observed in samples grown in LB compared to that of samples grown in BHI and TSB. This sample-level stability and variability was quantitated using Euclidean distances and plotted as matrices (Supplementary Figures 4–6) to clearly illustrate the sample-, strain- and species- level volatilomic differences. Hierarchical clustering coupled with heatmaps were also employed to analyze the similarities across the whole volatilomes of these bacterial samples. These plots are available in Supplementary Figures 1–3. In these heatmaps, samples were clustered based off Euclidean distance (dissimilarity). In these figures, to illustrate what compounds were responsible for the clustering of the bacterial samples, across the different media, hierarchical clustering was also performed on the compound abundances. Across the three media, the bacterial samples were generally successfully clustered to their respective species. There were some exceptions: EC.B_TSB_E in TSB and PA.A_LB_E in LB were incorrectly clustered; and some *E. coli* samples in LB formed a secondary E. coli cluster, this can also be seen in the PCA plot (Figure 1). These volatilomic differences between the E. coli samples in LB can be clearly seen in the heatmap shown in Supplementary Figure 3 and appear to be due to differences in the emission of accessory compounds. The results shown in the PCA plots (Figure 1), hierarchical clustering heatmaps (Supplementary Figures 1–3) clearly demonstrate that same compounds were responsible for the discrimination of the examined bacterial volatilomes across the growth media.

### Chemical Composition of Bacterial Volatilomes

**FIGURE 2:**
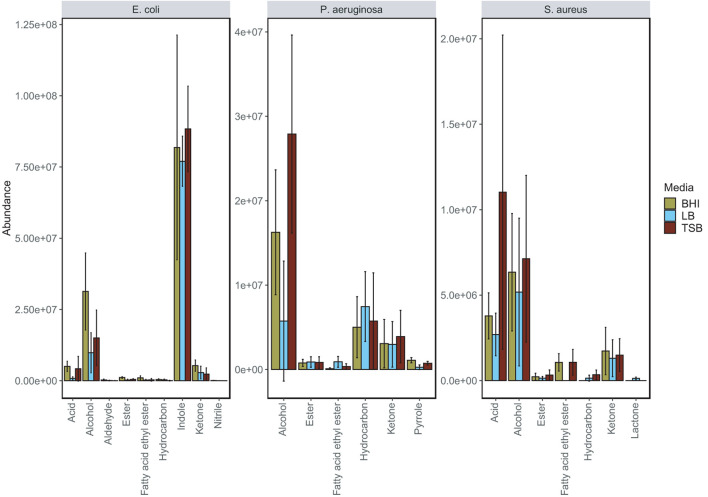
Grouped bar plot illustrating the differences in emission of individual chemical classes in BHI, LB and TSB growth media by *E. coli*, *P. aeruginosa* and *S. aureus*. This bar plot was obtained by summing the mean abundance of each chemical class detected in each of the examined bacteria. Significant media-dependent differences are illustrated in the corresponding grouped boxplot (Supplementary Figure 13).

The bar plots shown in Figure 2 illustrate the difference in abundance of each chemical class in BHI, TSB, and LB media for the species. Across each of the examined species it can be seen that for the majority of chemical classes, the lowest abundances of compounds were detected in LB media. Across the three nutrient-rich growth media, overall, there were not major variations in the chemical composition of the bacterial VOC profiles, and in this regard, were considered relatively stable. The results do suggest that for the three media examined, the bacterial volatilomes were species-dependent rather than media-dependent. Additional information about each individual compound identified can be found in the boxplots provided in Supplementary Figures 7–12.

**FIGURE 3:**
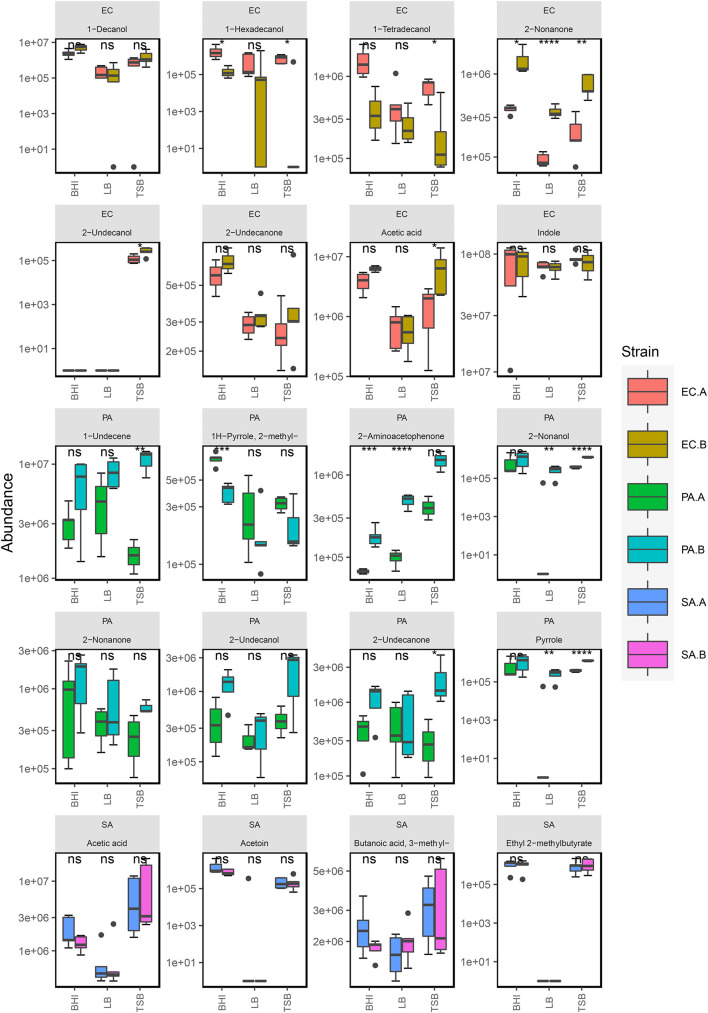
Box plot representation of selected compounds emitted by *S. aureus*, *P. aeruginosa*, and *E. coli* strains in BHI (*n* = 5), TSB (*n* = 5), and LB media (*n* = 5). *Five replicates were analyzed for each strain in each media except for *E. coli* (EC.A and EC.B) in BHI (*n* = 4) and *P. aeruginosa* (PA.B) in TSB (*n* = 3). The following symbols were used to indicate statistical significance of strain-level differences (ns: *p* > 0.05; *p ≤ 0.05; **p ≤ 0.01; ***p ≤ 0.001; ****p ≤ 0.0001). The corresponding strain names to the abbreviated titles of the bacterial samples shown in this plot are as follows: EC.A: *E. coli* DSM103372, EC.B: *E. coli* DSM30083, PA.A: *P. aeruginosa* DSM105372, PA.B: *P. aeruginosa* DSM25642, SA.A: *S. aureus* DSM2569, SA.B: *S. aureus* DSM799.

*E. coli* produced the highest number of VOCs in all media and was highly active metabolically in nutrient-rich environments as it emitted a diverse volatilome in all media. Across all of the media was mainly characterized by the heavy emission of indole, which was up to 500-fold more abundant than all other compounds in the volatilome. The grouped bar plots (Figure 2) and box plots (Figure 3) show that this hyper-emission was relatively uniform across the different media and confirm that this compound is an essential byproduct of *E. coli* metabolism. Other major chemical classes emitted by the *E. coli* strains were alcohols, acids, and ketones. These chemical classes did vary across the media as the abundance of 1-alcohols was relatively lower in LB compared to the BHI and TSB. Interestingly, in the majority of compounds, the emission of various ketones was slightly higher in LB samples compared to the BHI and TSB samples. This could be due to the higher dependence on the fatty acid metabolic pathway for energy rather than the primary metabolism of glucose that gives rise to a wide variety of acids, alcohols, and fatty acid ethyl esters. In contrast to *S. aureus*, the lack of glucose in the LB medium had a significant influence (Supplementary Figure 13) on the acid profile of the *E. coli* samples as the abundance of acetic acid saw up to a 12-fold reduction compared to samples cultured in TSB and BHI (Figure 3).

The primary chemical classes recovered in the *P. aeruginosa* strains were alcohols, ketones, hydrocarbons and pyrroles. Across the three media, *P. aeruginosa* emitted a variety of alcohols including 2-nonanol, 2-undecanol and 3-methyl-1-butanol, which were among the most abundant compounds produced (Figure 3). High abundances of alkene were emitted by both strains across all of the examined media highlighting it as one of the integral components of the *P. aeruginosa* volatilome. A significant reduction (Supplementary Figure 13) in alcohol abundance was observed in the samples cultured in LB medium. Higher abundances of 1-undecene were detected in *P. aeruginosa* LB samples. The chemical composition of the *P. aeruginosa* VOC profile in BHI and TSB is dominated by alcohols (Figure 2), this was the most radical media-induced shift that was observed. A possible explanation for this is that due to the lack of glucose in LB medium, fatty acid metabolic pathways were alternatively utilized to give rise to a relatively higher abundance of compounds such as 1-undecene. Pyrrole-like compounds were emitted by both strains in all media, with the highest abundances being emitted by the samples cultured in BHI medium (Figures 2, 3). The characteristic amine-containing ketone, 2-aminoacetophenone, was also emitted to varying degrees by both strains of *P. aeruginosa* (Figure 3). These results demonstrate the dual nature of the *P. aeruginosa* volatilome, as across different media, it exhibits stability on a qualitative level while exhibiting high variation on a quantitative level.

Across the examined media, 80% of the chemical composition of the observed *S. aureus* VOC profiles were acids and alcohols (Figure 2). Key compounds within these chemical groups were 3-methylbutyric acid, acetic acid, acetoin and 3-methyl-1-butanol (Figure 3). The relationship between acids and alcohols did however, vary across the three media, this can be seen in the grouped bar plots (Figure 2). In LB medium, overall acid abundance was reduced, for example, we observed a threefold reduction in acetic acid abundance between LB and BHI, and a 10-fold reduction between LB and TSB. Similarly, the emission of the majority of chemical classes by *S. aureus* samples was lowest in LB medium, primarily due to the lack of available glucose in the media. The influence of glucose on *S. aureus* volatilomes was also illustrated through the low abundances of key acids emitted in glucose-free nutrient broth (NB) (Nutrient Broth—Supplementary Figure 14). Less abundant chemical classes such as fatty acid ethyl esters, lactones, hydrocarbons and aldehydes demonstrated high variation across the media. In LB medium, low abundances of the closely associated compounds 1,4-butanediol and butyrolactone were recovered from both *S. aureus* strains—these compounds were not detected in BHI or TSB *S. aureus* samples. Conversely, butanoic acid, 2- methyl-, ethyl ester (ethyl 2-methylbutyrate) was emitted in relatively high abundances in BHI and TSB but wasn’t emitted in LB medium (Figure 3). These results further illustrate the significant influence that glucose has on the *S. aureus* volatilome. Additional information about all of the compounds identified across all the strains can be accessed in Supplementary Figures 8–12.

### Strain-Dependent Differences in Volatile Organic Compound Emission

**FIGURE 4:**
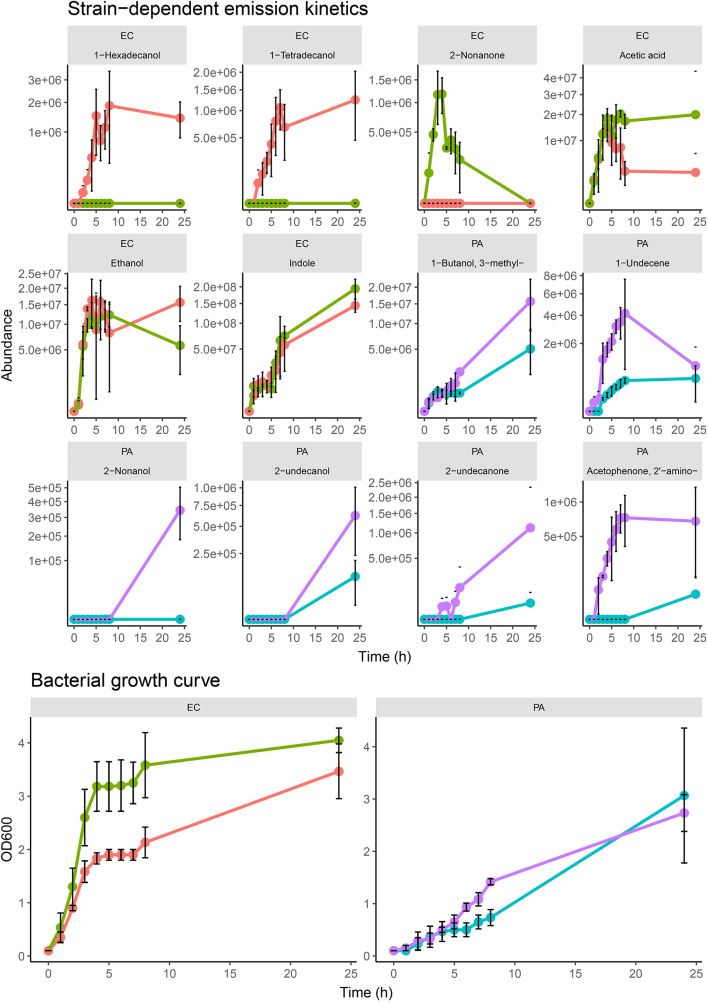
Kinetic profiles of strain-dependent emission from EC.A, EC.B, PA.A, and PA.B (for all examined strains, *n* = 3) for selected compounds sampled at specific phases of growth of bacterial samples in TSB growth media. Error bars represent the standard deviation around the mean abundance values. Y-axis labels on all VOC kinetic plots are scaled by square root. Corresponding cell growth curves based on OD_600_ measurements measured from each replicate at the respective sampling time. The corresponding strain names to the abbreviated titles of the bacterial samples shown in this plot are as follows: EC.A: *E. coli* DSM103372, EC.B: *E. coli* DSM30083, PA.A: *P. aeruginosa* DSM105372, PA.B: *P. aeruginosa* DSM25642.

Across the different growth media, we observed measurable differences in the emission of particular compounds between the strains of *P. aeruginosa* and *E. coli*. In Figure 3, compounds emitted by *E. coli* strains that had strain-dependent variation included 1-hexadecanol (*p* = 0.005 in TSB, *p* = 0.05 in BHI, *p* = 0.34 in LB), 1-tetradecanol (*p* = 0.003 in TSB, *p* = 0.01 in BHI, *p* = 0.14 in LB), acetic acid (*p* = 0.02 in TSB, *p* = 0.04 in BHI, *p* = 0.31 in LB), and 2-nonanone (*p* = 0.002 in TSB, *p* = 0.05 in BHI, *p* = 0.0001 in LB). In *P. aeruginosa* samples, the abundances of 1-undecene (*p* = 0.01 in TSB, *p* = 0.05 in BHI, *p* = 0.02 in LB), 2-aminoacetophenone (*p* = 0.008 in TSB, *p* = 0.0003 in BHI, *p* = 0.000005 in LB), 2-nonanol (*p* = 0.0003 in TSB, *p* = 0.193 in BHI, *p* = 0.008 in LB), 2-nonanone (*p* = 0.01 in TSB, *p* = 0.18 in BHI, *p* = 0.10 in LB), 2-undecanone (*p* = 0.09 in TSB, *p* = 0.01 in BHI, *p* = 0.33 in LB), and 2-undecanol (*p* = 0.10 in TSB, *p* = 0.06 in BHI, *p* = 0.33 in LB) showed a variety of differences between the two strains. The differences were consistent across the samples from each examined media indicating that these emission differences were due to strain-level specificity in VOC emission. To further investigate this, we analyzed the two *E. coli* and *P. aeruginosa* strains (*n* = 3) individually at progressive points in their growth in TSB medium to map the emission kinetic profile of these compounds and to ultimately determine if these strain-specific differences were consistent at varying time points. Growth curves for each strain were also constructed based on OD_600_ measured from the same samples (Figure 4). Of the aforementioned compounds, clear kinetic differences were observed in the compounds shown in Figure 4. The volatilomes of the two *S. aureus* strains were highly stable with respect to each other and therefore will not be discussed in this section.

In both *E. coli* strains, hyperproliferation of cells was observed between 0–4 h which corresponded to a proportionate emission of acetic acid, alcohols, and indole (Figure 4). The growth rate of EC.B cells was faster than that of EC.A. Of the alcohols emitted, 1-hexadecanol and 1-tetradecanol were only emitted by EC.A (Figure 4). Although these compounds were previously recovered from some of the EC.B samples in the previous investigation (Figure 3), they were emitted in relatively low abundances and there was variation in their occurrence sample-to-sample. Conversely, 2-nonanone was emitted in significantly higher abundances by EC.B than EC.A across the three growth media (*p* = 0.002 in TSB; *p* = 0.049 in BHI; *p* = 0.0001 in LB) (Supplementary Figure 4). This strain-dependent difference was further confirmed by the kinetic profiles of both strains (Figure 4), as 2-nonanone was not recovered from any EC.A samples while being emitted proportionately with the growth of EC.B cells (1–4 h). Cell growth of both strains stagnated for approximately 4 h (between 4–8 h), this stagnation was reflected by an overall reduction in the emission of VOCs from both strains between 5–6 h. From 8–24 h, cell numbers of both *E. coli* strains steadily grew again, however, abundances of compounds such as 1-alcohols collectively declined to varying degrees in both strains. Significantly high abundances of indole were emitted by both strains and correlated with the incubation time and growth of the cells.

In contrast to the growth of *E. coli* cells, both *P. aeruginosa* strains demonstrated a slower increase over the first 8 h of incubation. There was a marked difference in the volatilome activity of both strains (Figure 4). In agreement with the multi-media results shown in Figure 3, the kinetic plots shown in Figure 4 demonstrate that PA.B was metabolically more active than PA.A. High sample-to-sample variance in the occurrence of 2-undecanol, 2-undecanone, 2-nonanol, and 2-aminoacetophenone in PA.A samples across the media (Figure 3) and kinetically (Figure 4) indicated that these compounds were irregular accessory compounds to the PA.A volatilome. In contrast to this, 2-aminoacetophenone was a correlative marker of progressive phases of cell growth in PA.B samples, whereas 2-nonanol, 2-undecanol, and 2-undecanone marked the latter phase of PA.B growth as they were emitted at some point between 8–24 h. Although 3-methy-1-butanol was an abundant correlative growth marker of both *P. aeruginosa* strains, it was emitted at a threefold higher abundance in PA.B samples between 8–24 h (*p* = 0.05 at 24 h). Clear differences in the emission of 1-undecene (*p* = 0.01 in TSB; *p* = 0.05 in BHI; *p* = 0.02 in LB) across the three media (Figure 3) were observed between the two *P. aeruginosa* strains. This was also observed in the kinetic experiments as in the first 8 h of incubation, the abundances of 1-undecene recovered from PA.B were consistently 10-fold higher than the abundances recovered from PA.A (*p* = 0.009 at 4 h, *p* = 0.009 at 5 h, *p* = 0.004 at 6 h, *p* = 0.02 at 7 h). After 8 h, in PA.B samples, 1-undecene abundances sharply decreased.

## Discussion

Microorganisms produce a diverse range of volatile metabolites that have different physico-chemical properties and biological activities. These volatile metabolites serve an important role in inter-species and inter-kingdom communication and are involved in both beneficial and deleterious interactions between microorganisms (Hoshi et al., 2002). Microbial VOCs have been proposed as potential biomarkers of disease (Elmassry and Piechulla, 2020) that can be non-invasively analyzed to support clinical workflows in the future. Clinical studies involving untargeted breath profiling analyses have demonstrated the potential discriminatory power and diagnostic potential of VOCs for diseases such as pneumonia (Schnabel et al., 2015), cystic fibrosis (Robroeks et al., 2010; Neerincx et al., 2016), tuberculosis (Zetola et al., 2017), and COVID-19 (Ruszkiewicz et al., 2020). It has also been well-established that microbial diversity at the species-level is associated with poor outcomes and longer durations of diabetic ulcer wounds (Abdulrazak et al., 2005). A recent study also reported that diversity at the strain-level of specific pathogenic species is a large contributor to infection severity (Kalan et al., 2019). Consequently, the development of a non-invasive rapid sampling platform potentially capable of discriminating microbes at the species- and strain-level is highly desirable in clinic settings. In the conclusion of our previous study (Fitzgerald et al., 2020), we outlined that our future work will involve the volatilomic analysis of wound samples to identify infection-specific markers. Early unpublished results of this work suggests that volatile markers detected in this study such as 3-methylbutyric acid, acetic acid, 3-methyl-1-butanol, propanoic acid and ethanol do heavily persist in samples obtained from severe wound infections. However, this work is currently ongoing and a higher number of samples are required from a varying spectrum of infected wounds to further support these early findings.

Volatilomic profiling of pure microbial cultures has played a critical role in identifying the cellular origins of metabolites associated with specific species and strains of pathogens. In this study we employed HS-SPME-GC-MS to investigate the stability of the volatilome of multiple strains of prominent wound-associated pathogens across different nutrient-rich growth media. Oxygen availability is expected to have a strong influence on volatile compound formation by bacteria as it dictates whether respiration or fermentation pathways are utilized for metabolism (Weisskopf et al., 2021). Each bacterial sample was made up to a total volume of 5 mL, leaving a 15 mL headspace above the culture. It is predicted that the bacteria utilized the available oxygen in this headspace to carry out aerobic respiration in the early stages of growth. The examined pathogens are capable of anaerobic and/or fermentation under specific conditions, and may be capable of shifting their metabolism from aerobic respiration in oxygen-exhausted conditions (Lemfack et al., 2020). It was expected that this occurred during the 24 h incubation and consequently the volatilomes reported in this study are the result of both aerobic and anaerobic metabolism. Strain-level differences that were observed in the examined bacterial volatilomes were further investigated by analyzing and comparing the volatilome of each strain at progressive phases of cell growth and development.

Dimension reduction methods such as principal component analysis (PCA) (Tait et al., 2013; Chen et al., 2016; Fitzgerald et al., 2020) and clustering methods such as hierarchical clustering (HC) (Bean et al., 2016; Fitzgerald et al., 2020) are frequently employed to identify species-specific trends across the bacterial volatilomic samples. PCA identifies species- and strain-specific volatilomic differences across the bacterial volatilomes and amplifies these differences by constructing new linear variables called principal components (PCs), along which the variation is maximal. The PCs can then be visualized using scores plots. Scores plots show inter-sample distances that clearly illustrate patterns across the data which can be used to identify groups that characterize the overall dataset (Ringnér, 2008). In this study we used these techniques to perform an unsupervised analysis of the volatiomes obtained from the examined strains across each growth media. The PCA scores plots shown in Figure 1 illustrate that across the media the examined bacterial volatilomes demonstrated similar differentiation from each other which allowed clear and consistent species-level discrimination. These scores plots also indicate a certain degree of stability of the whole volatilomes of the examined species across the media. Similar volatilomic stability across different media was previously demonstrated by Dryahina et al. (2016)—in this study, PCA analysis of SIFT-MS volatilomic data showed that *S. aureus* and *P. aeruginosa* samples cultured in BHI, NB and Mueller Hinton Broth (MHB) exhibited species-dependent clustering rather than media-dependent clustering. Hierarchical clustering is another common statistical method used to classify multiple samples into groups (clusters). The results are visualized as dendrograms. The length of an edge in a dendrogram between a cluster and its split is proportional to the dissimilarity (Euclidean distance) between the split clusters (Wittek, 2014). Dendrograms were used for two purposes in this study, to investigate the volatilomic similarities between the examined bacterial strains, and to determine what compounds were responsible for the discrimination of the bacterial volatilomes across the different media. Heatmaps coupled with dendrograms complement the PCA results (Figure 1) and demonstrate clear discrimination of the bacterial species in each of the examined media (Supplementary Figures 1–3). The heatmaps also offer minor insights at the strain-level differences that are present in the volatilomes as the samples from *P. aeruginosa* (Supplementary Figures 1, 2) are roughly clustered together. In each of the examined media, it appeared that the same compounds were responsible for the discrimination of the bacterial volatilomes (Supplementary Figures 1–3). Following the analyses, the chemical classes and compounds responsible for the observed media-, species- and strain-dependent differences were further investigated.

Although the chemical compositions of each bacterial species demonstrated a high degree of stability (Figure 2), the abundances of chemical classes varied across BHI, TSB and LB growth medium (Figure 2). The volatilomes of both *E. coli* strains were dominated primarily by the emission of indole and fatty alcohols (collectively ∼ 85%), with the remaining 15% of the volatilome being mostly acids and ketones (Figure 2). Indole is produced in a one-step reaction by the enzymatic catalysation of the amino acid tryptophan (Lemfack et al., 2020). Growth media free of glucose such as LB medium contain an abundance of amino acids (Sezonov et al., 2007) that can be alternatively metabolized to form indole which explains why indole abundances were relatively uniform across the media (Figure 2). *E. coli* primarily produces fatty alcohols *via* the fatty acid metabolic pathways (Liu et al., 2016), however, fatty alcohols can also be derived from glucose metabolism (Youngquist et al., 2013). A variety of fatty alcohols were identified at varying abundances across the examined media (Figure 3 and Supplementary Figure 5) between the two *E. coli* strains. Despite EC.B cells having a higher growth rate, EC.A emitted significantly higher abundances of 1-tetradecanol and 1-hexadecanol than EC.B across the media (Figure 3) and temporally (Figure 4). This supports the fact that the emission of these compounds is not just simply dependent on the progressive growth of cells, but rather the different metabolic pathways each strain utilizes as the cells proliferate. Fatty alcohols (C_3_–C_16_) have been frequently reported (van den Berg et al., 2006; Bos et al., 2013; Chen et al., 2016; Fitzgerald et al., 2020) to be emitted by *E. coli* with varying combinations being emitted strain-to-strain. Acetic acid abundances were high in both strains (Figure 3) and demonstrated a proportionate increase with cell proliferation in the first 5 h of incubation (Figure 4).

*P. aeruginosa* is a common gram-negative bacteria that has been associated with severe infections of burns (Lyczak et al., 2000) and diabetic foot ulcers (Abdulrazak et al., 2005), and it has also been previously labeled as the most common cause of persistent, fatal respiratory infections in cystic fibrosis patients (Bradbury, 2009; Davis et al., 2020). The chemical composition of the observed *P. aeruginosa* volatilomes was primarily made up of alcohols and hydrocarbons (∼75%) across the media (Figure 2). This appears to be in agreement with recent results published by Davis et al.(Scott-Thomas et al., 2010), although there are differences in the *P. aeruginosa* volatilomes at the compound-level. The remaining 25% of the volatilomes were composed of ketones, pyrroles and fatty acid esters (Figure 2). Characteristic compounds (Figure 3) emitted by both strains across the three media that have also been frequently detected in *P. aeruginosa* volatilomic analyses included 2-aminoacetophenone (Scott-Thomas et al., 2010; Shestivska et al., 2012; Tait et al., 2013), 2-undecanol (Filipiak et al., 2012b; Fitzgerald et al., 2020), 2-undecanone (Tait et al., 2013; Timm et al., 2018; Fitzgerald et al., 2020), 1-undecene (Filipiak et al., 2012b; Shestivska et al., 2012; Tait et al., 2013; Fitzgerald et al., 2020), 2-nonanone (Scott-Thomas et al., 2010; Filipiak et al., 2012b; Fitzgerald et al., 2020), and pyrrole (Filipiak et al., 2012b; Tait et al., 2013; Fitzgerald et al., 2020). Production of 1-undecene by both strains of *P. aeruginosa* was enhanced when the samples were cultured in LB medium most likely due to the absence of glucose (Figures 2, 3). Although significant differences in 1-undecene emission were observed between both strains across the media (Figure 3) and kinetically (Figure 4), 1-undecene was shown to be a temporal growth marker of both strains. This long chain alkene is derived from the metabolism of fatty acids and is a key component of the volatilomes of various *Pseudomonas spp.* (Dandurishvili et al., 2010; Kanchiswamy et al., 2015; Lo Cantore et al., 2015). 2-aminoacetophenone is produced in the amino acid degeneration (shikimate) pathway *via* the loss of a hydroxyl group on anthranilic acid (Weisskopf et al., 2021) [derived from chorismate *via* chorismate lyase (Parthasarathy et al., 2018)]. Despite being widely reported as a characteristic marker for *P. aeruginosa* (Shestivska et al., 2012), we observed a high degree of strain-dependent variation in both its emission across the different media (Figure 3) and kinetically (Figure 4). Our results suggest that the amino acid degradation pathway may potentially be more active in some *P. aeruginosa* strains than others. Differences in 2-aminoacetophenone production could also be due to strain-level differences in the regulation of quorum sensing (Kesarwani et al., 2011).

Since the emergence of antibiotic-resistant strains of *S. aureus* that caused epidemics in the 1950s and 1960s there have been large global efforts to develop early detection systems (Cookson, 2011). In the last 15 years, the understanding of the core and accessory components of the *S. aureus* volatilome has been steadily growing due to a growing number of *in vitro* volatilomic profiling studies (Bunge et al., 2008; Filipiak et al., 2012b; Baptista et al., 2019; Fitzgerald et al., 2020; Jenkins and Bean, 2020). The clinical value of *in vitro* volatilomic profiling has been demonstrated by Filipiak et al. (2015) who reported detection of VOCs, known to be emitted by *S. aureus*, in the breath of patients with *S. aureus*-positive respiratory infections. In this study here, the *S. aureus* volatilome demonstrated the highest degree of stability across the media out of the three examined species. This was illustrated by the tight clusters of “SA” samples in each score plot in Figure 1 and in the heatmaps (Supplementary Figures 1–3). Across TSB, BHI and LB media, the *S. aureus* volatilome was composed primarily of acids and alcohols (∼80% total volatilome), at different ratios in each. Other chemical classes recovered in all media included ketones and fatty acid esters. The characteristic compounds that were emitted by both *S. aureus* strains were not surprising and included 3-methylbutyric acid, acetic acid, ethyl 2-methylbutyrate and acetoin—all of which have been frequently reported in the literature (Filipiak et al., 2012b, 2015; Chen et al., 2016; Jenkins and Bean, 2020). These key compounds all originate from primary metabolic pathways (Weisskopf et al., 2021). 3-methylbutyric acid is derived from the metabolism of amino acids (leucine) while acetic acid, ethyl 2-methylbutyrate and acetoin arise from different stages of the fermentation process in glucose metabolism (Schulz et al., 2020; Weisskopf et al., 2021). The different primary metabolic pathways from which these compounds are derived are reflected in Figure 3, as in the glucose-free LB medium, acetic acid, ethyl 2-methylbutyrate and acetoin abundances are significantly lower than abundances observed in TSB and BHI while 3-methylbutyric acid emission was comparable across the media due to presence of amino acid substrates in all media. Similar reductive effects on the emission of acids and esters by *S. aureus* in glucose-free media was recently reported by Jenkins and Bean (2020).

Comprehensive bacterial volatilomic data will only be obtained through the analysis of a high number of strains. Strain-to-strain volatilomic variability has been relatively under studied, however, studies that have been carried out have highlighted potential strain-specific differences in bacterial volatilomes. Purcaro et al. (2019) reported discrimination of multiple strains of *P. aeruginosa* in the breath of infected mice. In our previous study (Fitzgerald et al., 2020), when analyzing triplicate samples of the same strains, we observed small differences between both *P. aeruginosa* and *E. coli* strains and concluded that further work is needed incorporating a higher number of samples to comprehensively resolve these differences. Analyzing different microbial strains at specific stages of cell growth and development has been recently proposed as an effective approach to comprehensively elucidate the variation in microbial volatilomes at the strain-level (Kai, 2020). In this study, we employed this approach in a more targeted manner to further investigate the strain-dependent emission variation of specific compounds—observed across multiple media—in *P. aeruginosa* and *E. coli* samples. The resulting kinetic plots shown in Figure 4 confirm that there was consistent strain-level specificity in the temporal emission of individual compounds. These results were in agreement with our earlier observed differences between the strains for the same compounds in Figure 3. The results obtained from this study also validate observed differences in VOC emission previously reported (Fitzgerald et al., 2020) between *P. aeruginosa* strains. Although the volatilomes obtained from all of the *E. coli* samples in this study are mostly in agreement with those previously reported (Fitzgerald et al., 2020), the same strain-level differences were not observed. It should also be noted, that when analyzing the full spectrum of VOCs recovered from these strains in an unsupervised manner *via* PCA (Figure 1), neither *P. aeruginosa* nor *E. coli* strains were discriminated from each other. Our results show that when investigating the full volatilomes of these strains, clear discrimination of the strains will not be achieved, it is only when the volatilomes are investigated at the compound level with corresponding emission kinetic data that these strain-level differences can be fully elucidated. While our data clearly shows strain-level volatilomic differences exist within specific species of bacteria, it also highlights the complexity of strain-level volatilomic discrimination. Strain-level differences were not observed between the examined *S. aureus* strains in this study, however, volatilomic discrimination of different *S. aureus* strains (MSSA and MRSA) has been previously reported (Boots et al., 2014) indicating that strain-dependent antibiotic sensitivities could also be potential factor in strain-level volatilomic diversity. Baptista et al. (2019) reported a high number of compounds and also demonstrated strain-level volatilomic differences across *S. aureus* samples based on enterotoxicity. Microbial volatilomic variability across different media has been frequently reported in the literature (Tait et al., 2013; Rees et al., 2017; Zareian et al., 2018; Jenkins and Bean, 2019). This was particularly highlighted by Rees et al. (2017) who detected a total of 365 compounds from 9 *Klebsiella pneumoniae* clinical isolates across TSB, LB, BHI and MHB media—only 10% of the compounds were common across all examined media. Many of the studies discussed in this text have highlighted the complexity and high degree of specificity of microbial volatilomes. Future work should focus on investigating the strain-level volatilomic diversity present within specific microbial species by analyzing a higher number of strains obtained from ecologically varying environments across different media. It is through, firstly, comprehensively investigating the factors that govern the emission of pathogen-specific metabolites that will allow volatilomics to be employed in clinics in the future.

## Conclusion

Bacterial volatilomes are influenced by different nutritional environments and strain-level differences. By investigating this stability in this work a comprehensive understanding of the volatilomes of the examined bacterial species was achieved. Our objectives in this study were: (1) to obtain comprehensive volatilomic data from multiple strains of the wound-associated pathogens *S. aureus, P. aeruginosa* and *E. coli* in three different growth media; (2) to assess the stability and variation of the observed bacterial volatilomes; and (3) to assess strain-level specificity within the examined strains by comparing the emission of specific compounds at progressive stages of growth and development of the cells. Using HS-SPME GC-MS analysis we successfully analyzed the VOCs produced from each strain to obtain characteristic species-specific volatilomes. The observed volatilomes demonstrated a high degree of stability across the examined media, however, glucose-free media had a reductive effect on the emission of various primary metabolites. Strain-level variation was observed in *P. aeruginosa* and *E. coli* samples across the examined media in the emission of particular compounds. Comparative temporal volatilomic analysis of these strains confirmed that there were differences in the emission of individual compounds between the examined strains. Moving forward in microbial volatilomics, performing similar multi-strain kinetic experiments will provide a more comprehensive view of the capabilities of microbial volatilomes. Additionally, analyzing microbial volatilomes in different growth media allows specific metabolic pathways responsible for VOC production to be intimately investigated. Building a comprehensive understanding of the limits and possibilities of microbial volatilomics will elucidate what can be ultimately achieved in future applications.

On a clinical level, these pathogens pose a significant challenge as they cause severe wound infections. We are currently working on the study of the diabetic foot ulcer volatilome and have seen that some characteristic compounds (e.g., 3-methyl-1-butanol, 3-methylbutyric acid, and ethanol) also persist in wound samples. Our clinical work is still in the early stages, however, we hope to identify infection-specific volatilomic patterns that will potentially allow the detection of wound infections in the early stages.

## Data Availability Statement

The datasets presented in this study can be found in online repositories. The names of the repository/repositories and accession number(s) can be found below: https://figshare.com/articles/dataset/Wound-associated_bacterial_pathogens_volatilomic_data/16692217.

## Author Contributions

SF performed the experimental work and drafted the manuscript. LH and AM supervised the work and reviewed the manuscript. All authors contributed to the article and approved the submitted version.

## Conflict of Interest

The authors declare that the research was conducted in the absence of any commercial or financial relationships that could be construed as a potential conflict of interest.

## Publisher’s Note

All claims expressed in this article are solely those of the authors and do not necessarily represent those of their affiliated organizations, or those of the publisher, the editors and the reviewers. Any product that may be evaluated in this article, or claim that may be made by its manufacturer, is not guaranteed or endorsed by the publisher.
