# Supplementary material for: An Investigation of Stability and Species and Strain-Level Specificity in Bacterial Volatilomes
Source: Front Microbiol. 2021 Oct 13;12:693075. doi: 10.3389/fmicb.2021.693075 (PMC8549763; doi:10.3389/fmicb.2021.693075)
Supplement: Supplementary file 1 [file Data_Sheet_1.pdf]

An investigation of stability and species and strain-level specificity in bacterial volatilomes – Supplementary Information

Shane Fitzgerald[1], Linda Holland[2], Aoife Morrin*[1]

1. School of Chemical Sciences, National Centre for Sensor Research, Insight SFI Research Centre for Data Analytics, Dublin City University, Ireland
2. School of Biotechnology, Dublin City University, Ireland

*aoife.morrin@dcu.ie

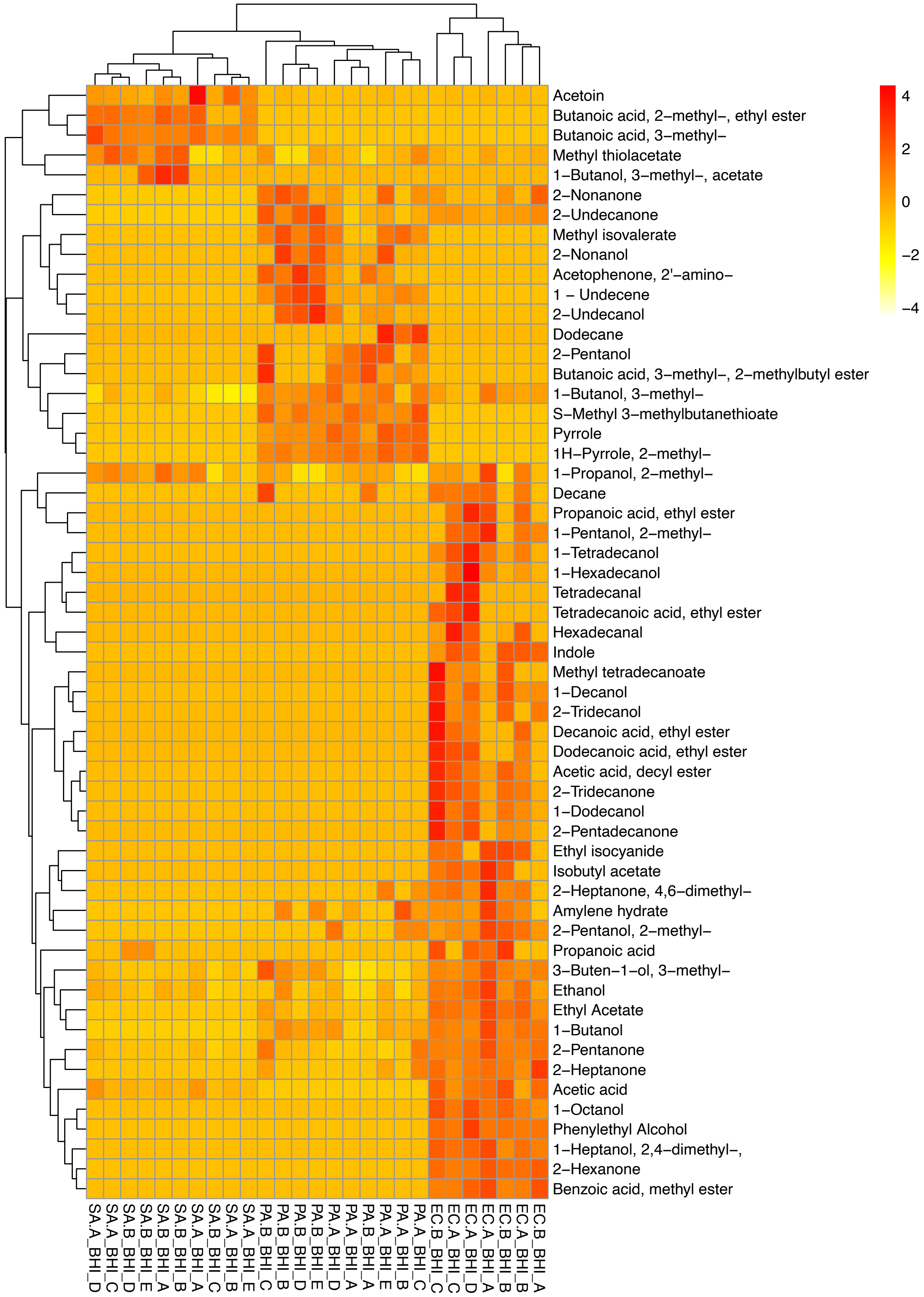

Figure S1: Heatmap showing the abundance of VOCs recovered (rows) from each bacterial strain (columns) cultured in BHI media. Values were scaled and centred by their respective rows, with highly abundant VOCs being coloured red, and less abundant VOCs being marked orange - yellow . Dissimilarity between the samples was measured using Euclidean distance.

The complete agglomeration method was used. The corresponding strain names to the abbreviated titles of the bacterial samples shown in this plot are as follows: ***EC.A:*** *E. coli DSM103372,* ***EC.B:*** *E. coli DSM30083,* ***PA.A****: P. aeruginosa DSM105372,* ***PA.B****: P. aeruginosa DSM25642,* ***SA.A:*** *S. aureus DSM2569,* ***SA.B:*** *S. aureus DSM799*

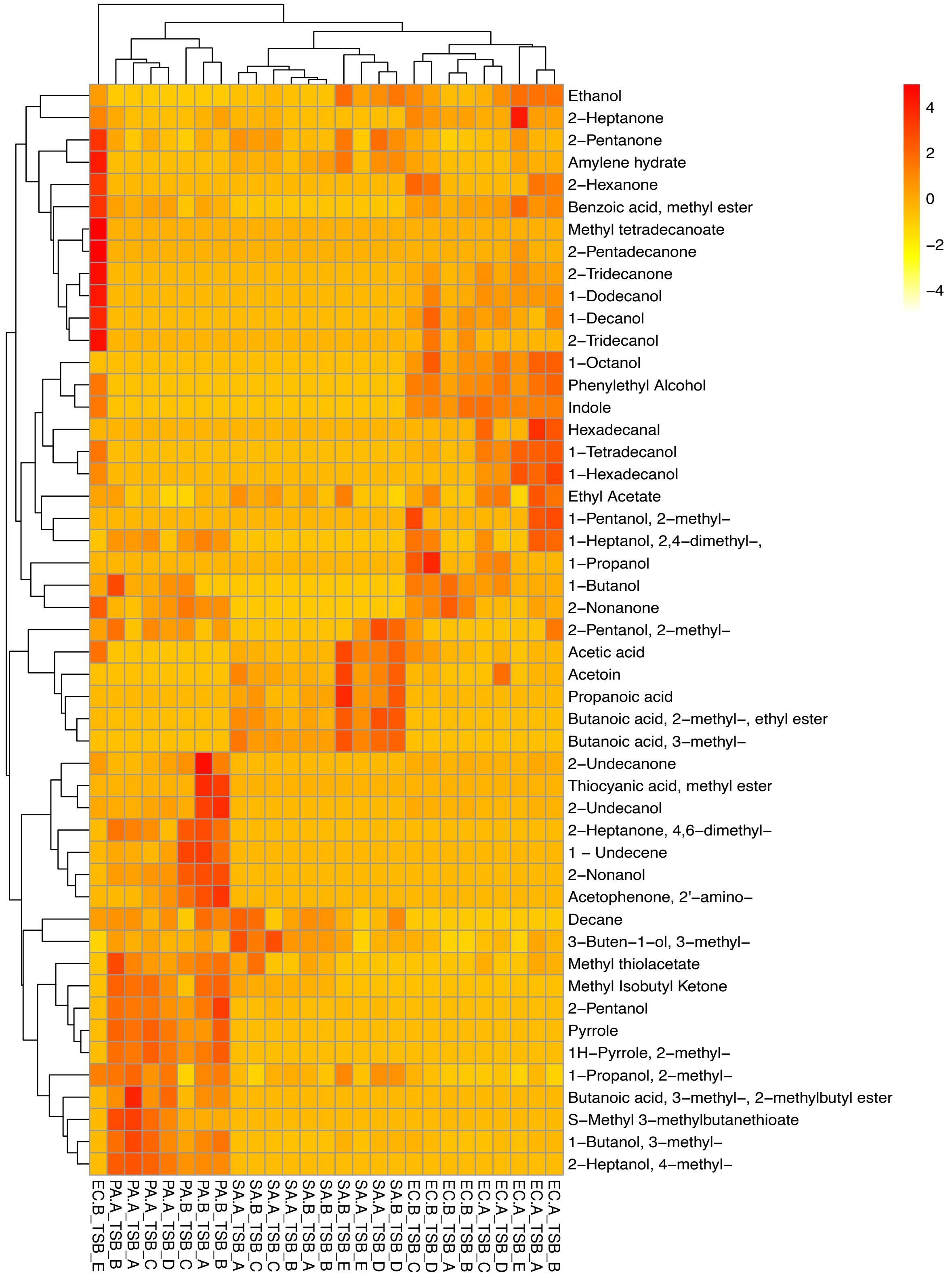

Figure S2: Heatmap showing the abundance of VOCs recovered (rows) from each bacterial strain (columns) cultured in TSB media . Values were scaled and centred by their respective

rows, with highly abundant VOCs being coloured red, and less abundant VOCs being marked orange - yellow . Dissimilarity between the samples was measured using Euclidean distance. The complete agglomeration method was used. The corresponding strain names to the abbreviated titles of the bacterial samples shown in this plot are as follows: ***EC.A:*** *E. coli DSM103372,* ***EC.B:*** *E. coli DSM30083,* ***PA.A****: P. aeruginosa DSM105372,* ***PA.B****: P. aeruginosa DSM25642,* ***SA.A:*** *S. aureus DSM2569,* ***SA.B:*** *S. aureus DSM799*

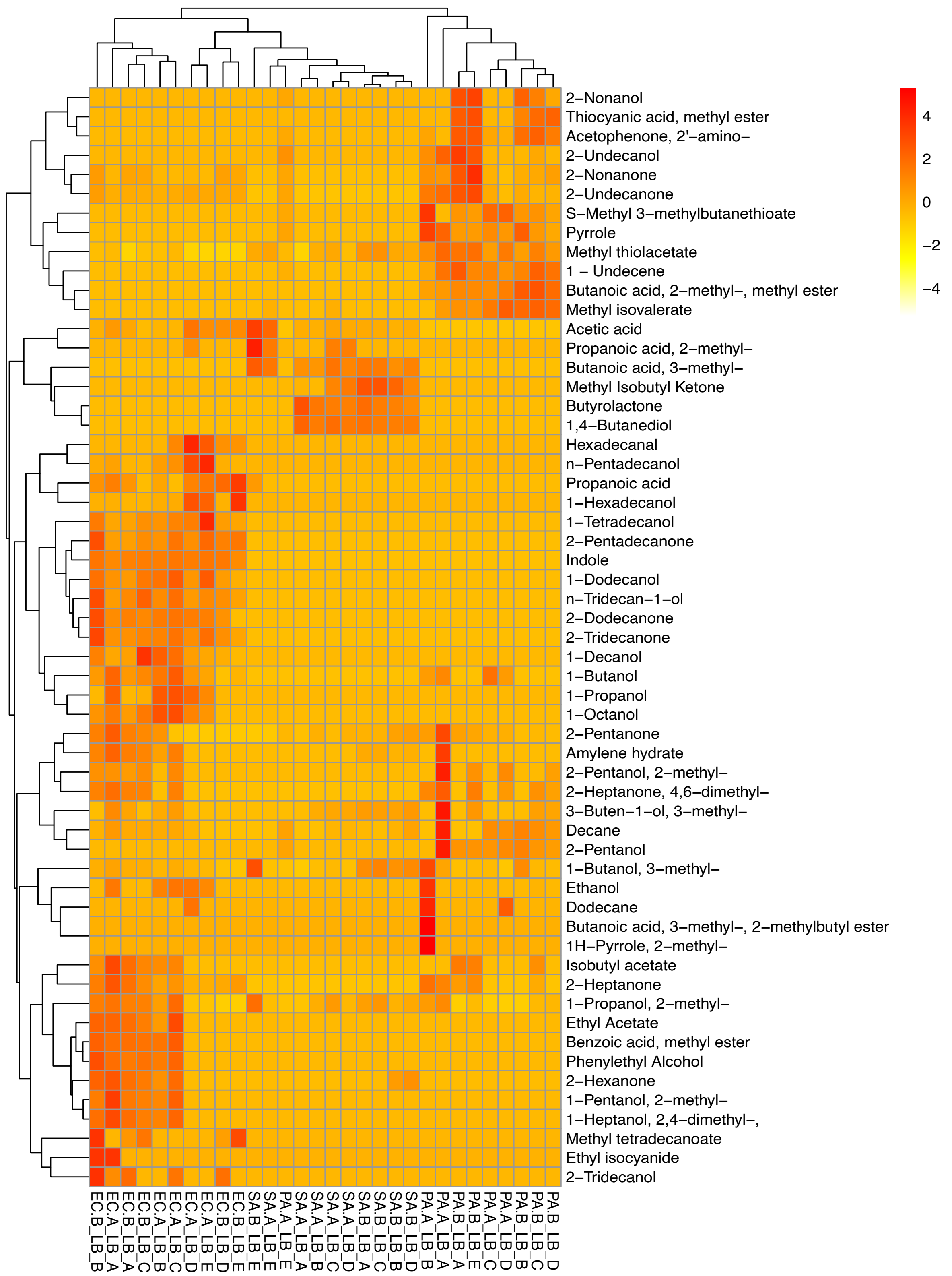

Figure S3: Heatmap showing the abundance of VOCs recovered (rows) from each bacterial strain (columns) cultured in LB media. Values were scaled and centred by their respective rows, with highly abundant VOCs being coloured red, and less abundant VOCs being marked orange - yellow. Dissimilarity between the samples was measured using Euclidean distance. The complete agglomeration method was used. The corresponding strain names to the abbreviated titles of the bacterial samples shown in this plot are as follows: ***EC.A:*** *E. coli*

DSM103372, ***EC.B:*** *E. coli DSM30083,* ***PA.A****: P. aeruginosa DSM105372,* ***PA.B****: P. aeruginosa DSM25642,* ***SA.A:*** *S. aureus DSM2569,* ***SA.B:*** *S. aureus DSM799*

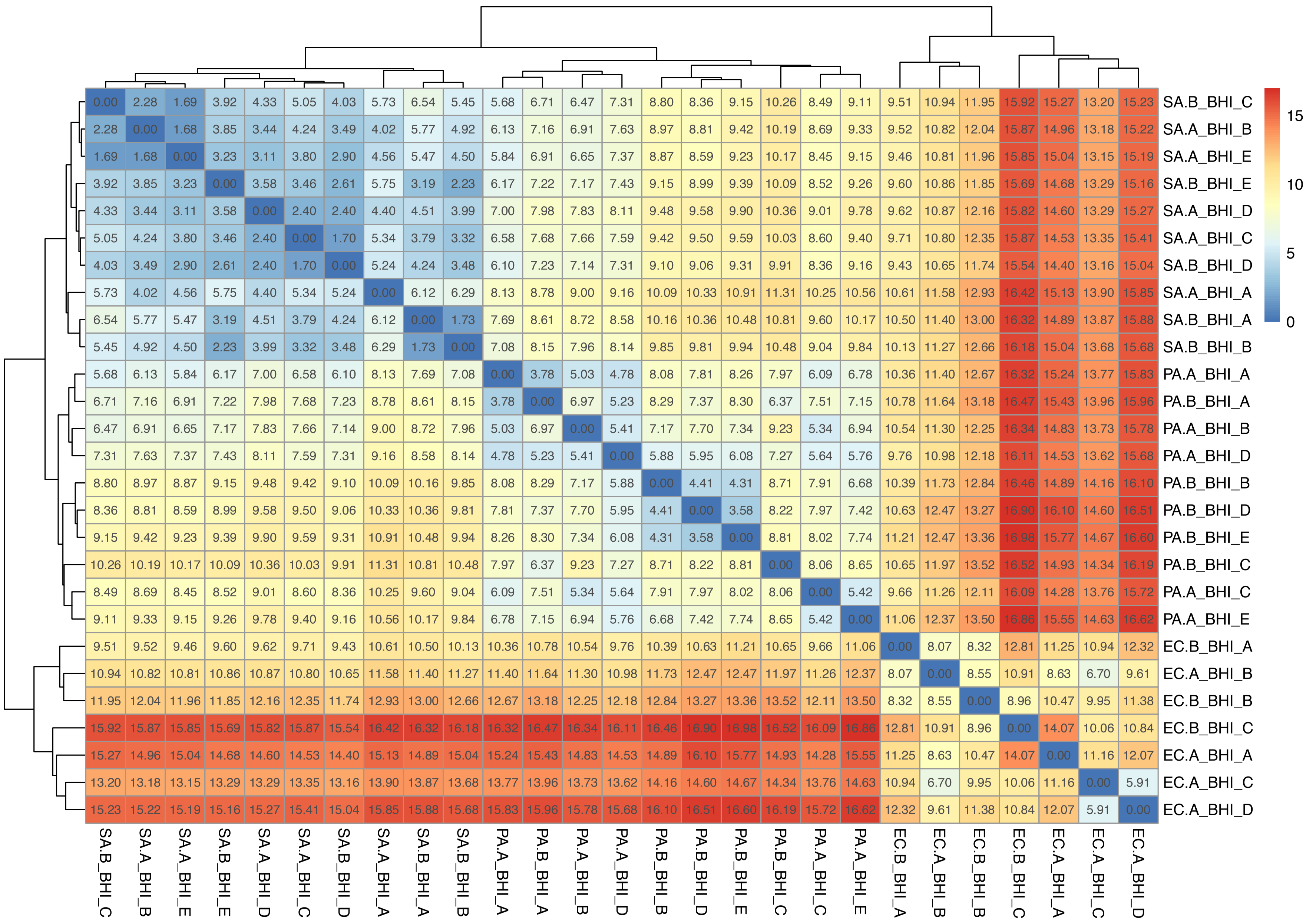

Figure S4: Heatmap dissimilarity matrix representation of bacterial samples cultured in BHI. Values are calculated Euclidean distances. Red represents highly dissimilar samples; blue represents highly similar samples. The corresponding strain names to the abbreviated titles of the bacterial samples shown in this plot are as follows: ***EC.A:*** *E. coli DSM103372,* ***EC.B:*** *E. coli DSM30083,* ***PA.A****: P. aeruginosa DSM105372,* ***PA.B****: P. aeruginosa DSM25642,* ***SA.A:*** *S. aureus DSM2569,* ***SA.B:*** *S. aureus DSM799*

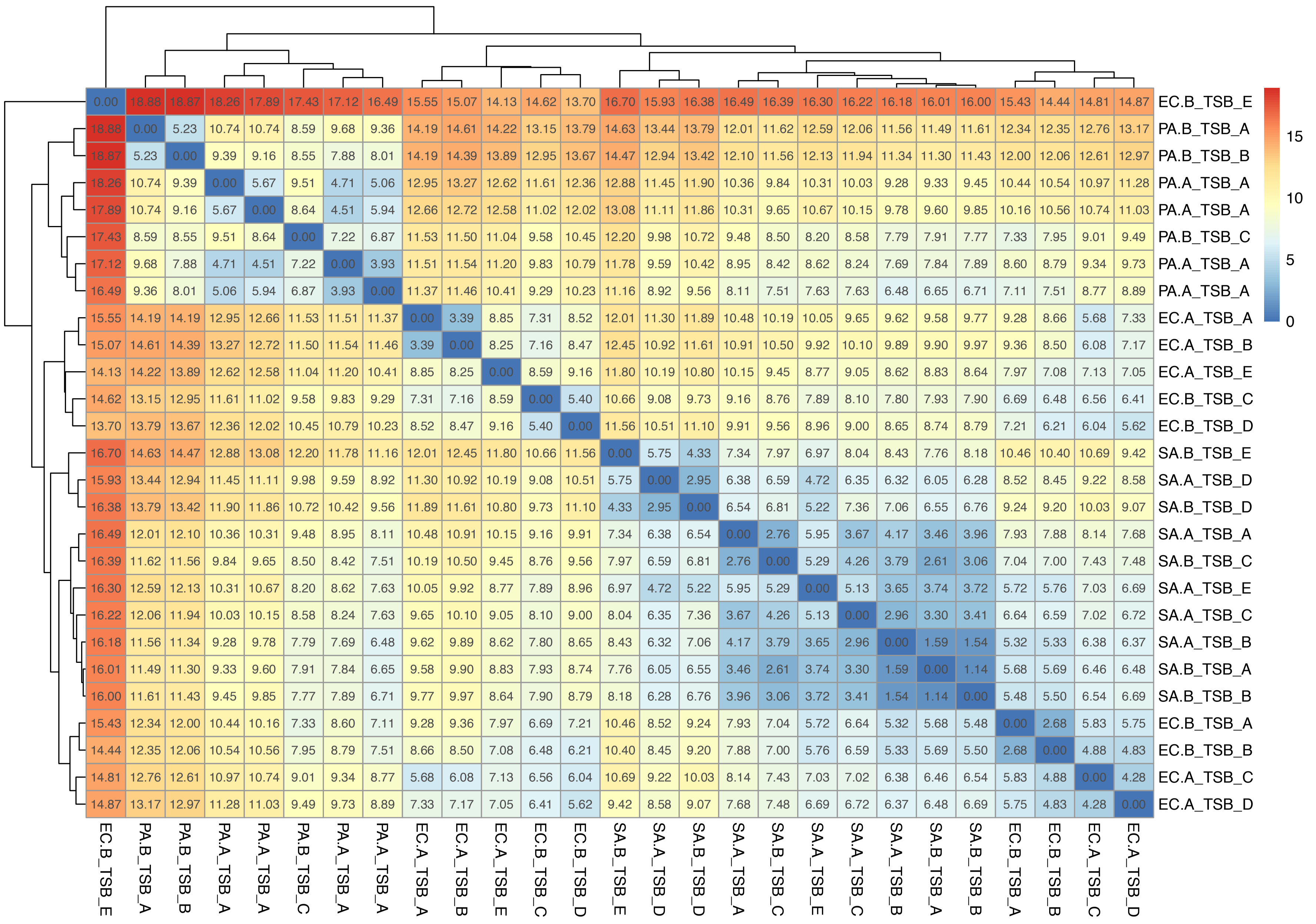

Figure S5: Heatmap dissimilarity matrix representation of bacterial samples cultured in TSB. Values are calculated Euclidean distances. Red represents highly dissimilar samples; blue represents highly similar samples. The corresponding strain names to the abbreviated titles of the bacterial samples shown in this plot are as follows: ***EC.A:*** *E. coli DSM103372,* ***EC.B:*** *E. coli DSM30083,* ***PA.A****: P. aeruginosa DSM105372,* ***PA.B****: P. aeruginosa DSM25642,* ***SA.A:*** *S. aureus DSM2569,* ***SA.B:*** *S. aureus DSM799*

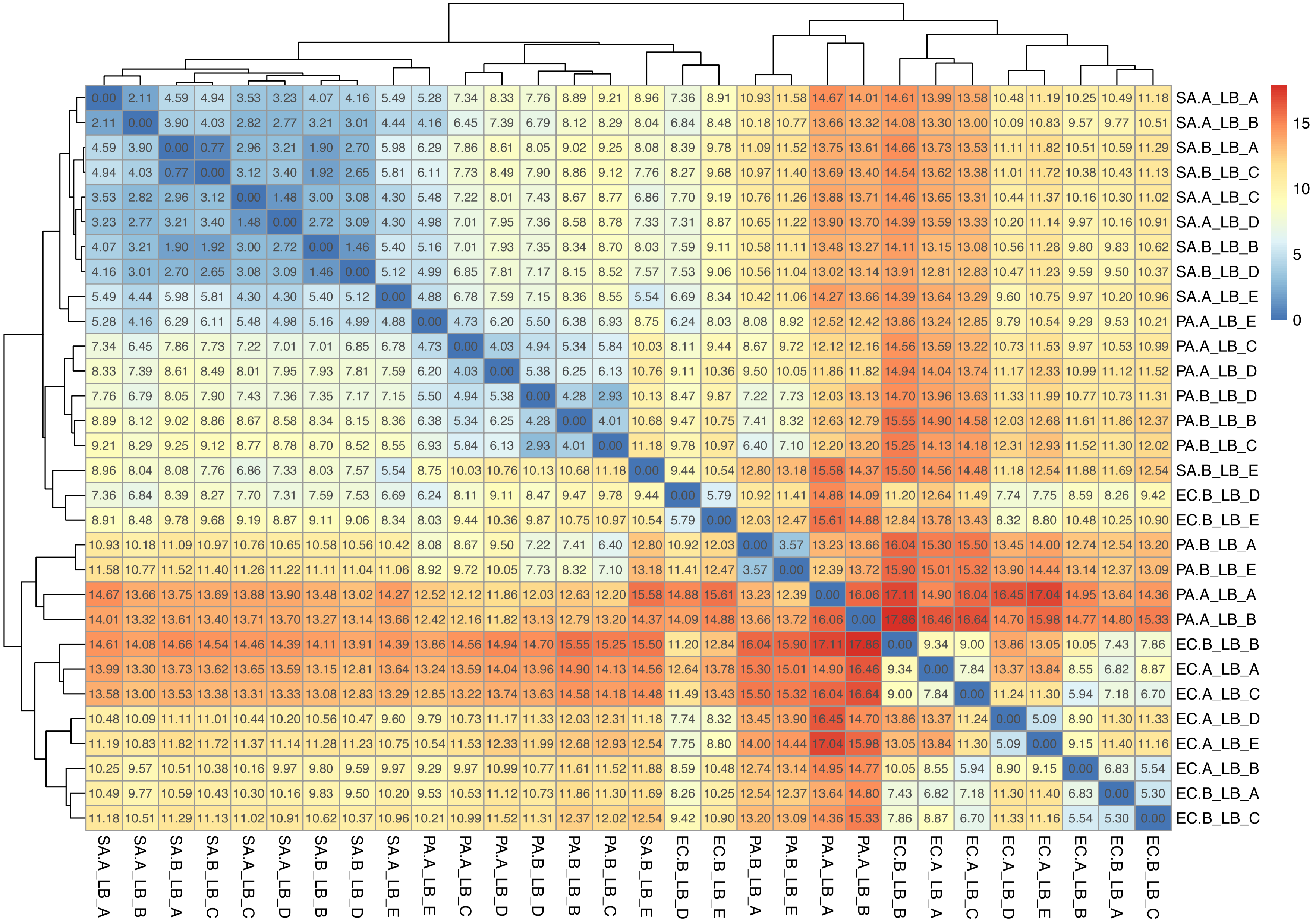

Figure S6: Heatmap dissimilarity matrix representation of bacterial samples cultured in LB. Values are calculated Euclidean distances. Red represents highly dissimilar samples; blue represents highly similar samples. The corresponding strain names to the abbreviated titles of the bacterial samples shown in this plot are as follows: ***EC.A:*** *E. coli DSM103372,* ***EC.B:*** *E. coli DSM30083,* ***PA.A****: P. aeruginosa DSM105372,* ***PA.B****: P. aeruginosa DSM25642,* ***SA.A:*** *S. aureus DSM2569,* ***SA.B:*** *S. aureus DSM799*

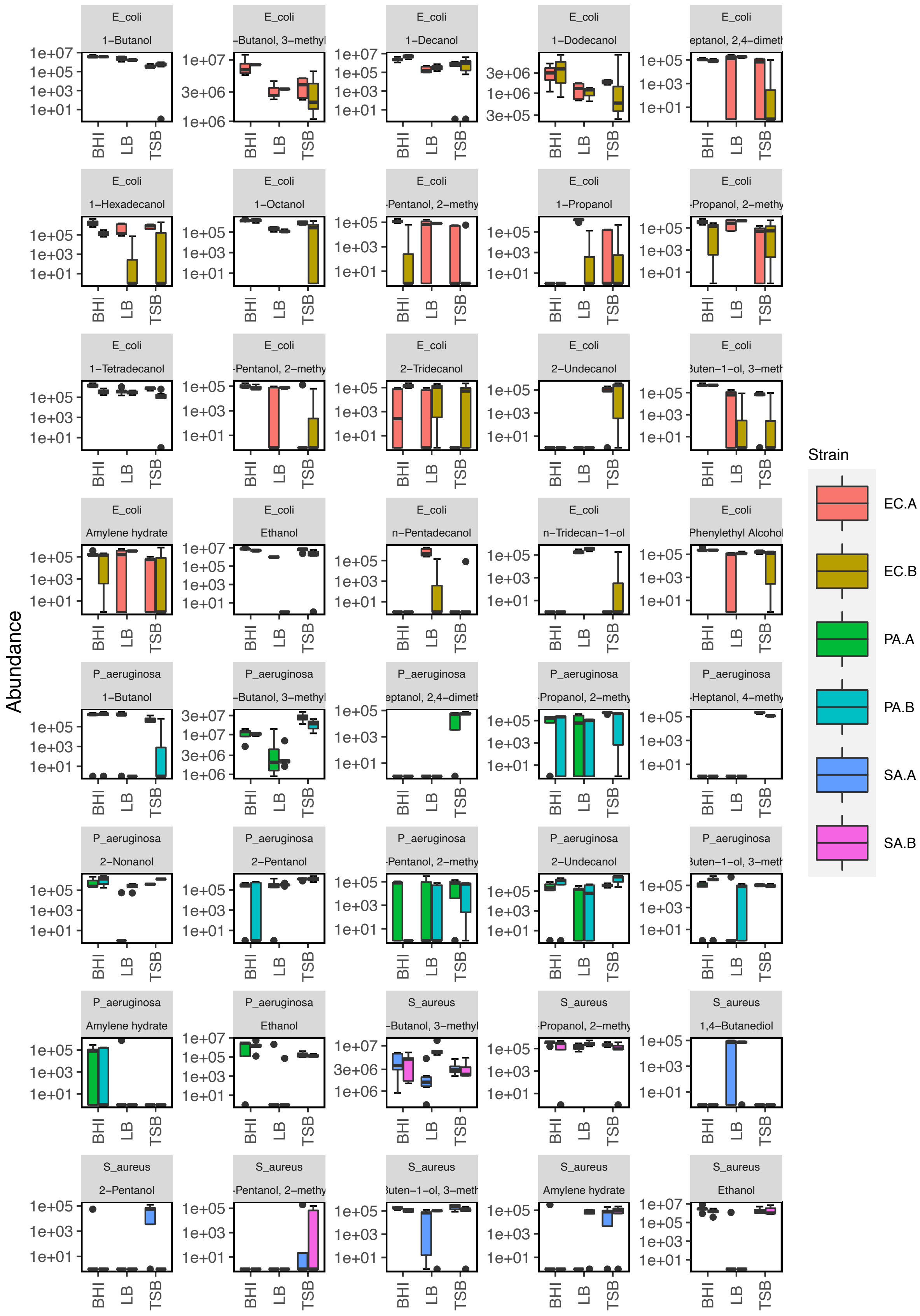
Abundance
Strain
EC.A
EC.B
PA.A
PA.B
SA.A
SA.B
BHI
LB
TSB
E_coli
1−Butanol
-Butanol, 3−methyl
1−Decanol
1−Dodecanol
eptanol, 2,4−dimeth
1−Hexadecanol
1−Octanol
-Pentanol, 2−methy
1−Propanol
-Propanol, 2−methy
1−Tetradecanol
-Pentanol, 2−methy
2−Tridecanol
2−Undecanol
3uten−1−ol, 3−meth
Amylene hydrate
Ethanol
n−Pentadecanol
n−Tridecan−1−ol
Phenylethyl Alcohol
P_aeruginosa
1−Butanol
-Butanol, 3−methyl
eptanol, 2,4−dimeth
-Propanol, 2−methy
-Heptanol, 4−methy
2−Nonanol
2−Pentanol
-Pentanol, 2−methy
2−Undecanol
3uten−1−ol, 3−meth
Amylene hydrate
Ethanol
S_aureus
-Butanol, 3−methyl
-Propanol, 2−methy
1,4−Butanediol
2−Pentanol
-Pentanol, 2−methy
3uten−1−ol, 3−meth
Amylene hydrate
Ethanol
1e+01
1e+03
1e+05
1e+07
1e+06
3e+06
3e+07
3e+05

Figure S7 : Species- and alcohol-specific boxplots. Each plot illustrates the abundance of an individual alcoholic compound emitted by two strains of a species (*E. coli, P. aeruginosa*, and *S. aureus*) across three nutrient-rich media (Brain Heart Infusion – BHI; Lysogeny Broth – LB; Tryptone Soy Broth – TSB). Each strain is colour-coded according to the legend at the left side of the plot. *For each examined strain in BHI, n=5;, TSB, n=5; and LB media, n = 5. *Five replicates were analysed for each strain in each media except for E. coli (EC.A and EC.B) in BHI (n = 4) and P. aeruginosa (PA.B) in TSB (n = 3). The following compound names are partially visible: row 1 , 2nd and 5th : 3-methyl-1-butanol and* 1-Heptanol, 2,4-dimethyl-; row 2, 3rd and 5th: 1-Pentanol, 2-methyl- and 1-Propanol, 2-methyl-; row 3, *2nd and 5th :* 2-Pentanol, 2-methyl- and 3-Buten-1-ol, 3-methyl-; row 5, 2nd, 3rd, and 5th : *3-methyl-1-butanol,* 1-Heptanol, 2,4-dimethyl-, 1-Propanol, 2-methyl-, and 2-Heptanol, 4-methyl-; row 6, 3rd and 5th : 1-Pentanol, 2-methyl and 3-Buten-1-ol, 3-methyl-; row 7, 3rd and 4th : *3-methyl-1-butanol and* 1-Propanol, 2-methyl-; row 8, 2nd and 3rd : 1-Pentanol, 2-methyl and 3-Buten-1-ol, 3-methyl-
.

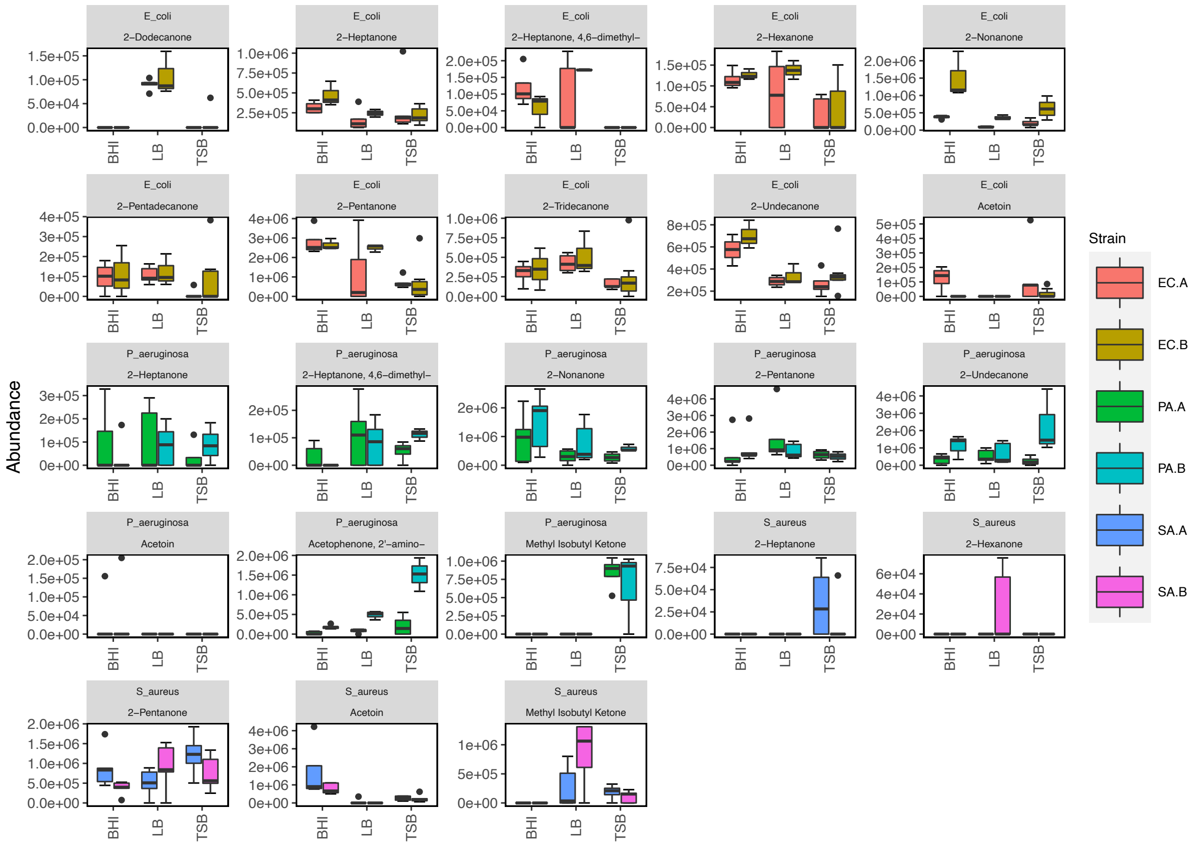

Figure S8 : Species- and ketone-specific boxplots. Each plot illustrates the abundance of an individual ketone compound emitted by two strains of a species (*E. coli, P. aeruginosa*, and *S. aureus*) across three nutrient-rich media (Brain Heart Infusion – BHI; Lysogeny Broth – LB; Tryptone Soy Broth – TSB). Each strain is colour-coded according to the legend at the left side of the plot. *For each examined strain in BHI, n=5;, TSB, n=5; and LB media, n = 5. *Five replicates were analysed for each strain in each media except for E. coli (EC.A and EC.B) in BHI (n = 4) and P. aeruginosa (PA.B) in TSB (n = 3).*

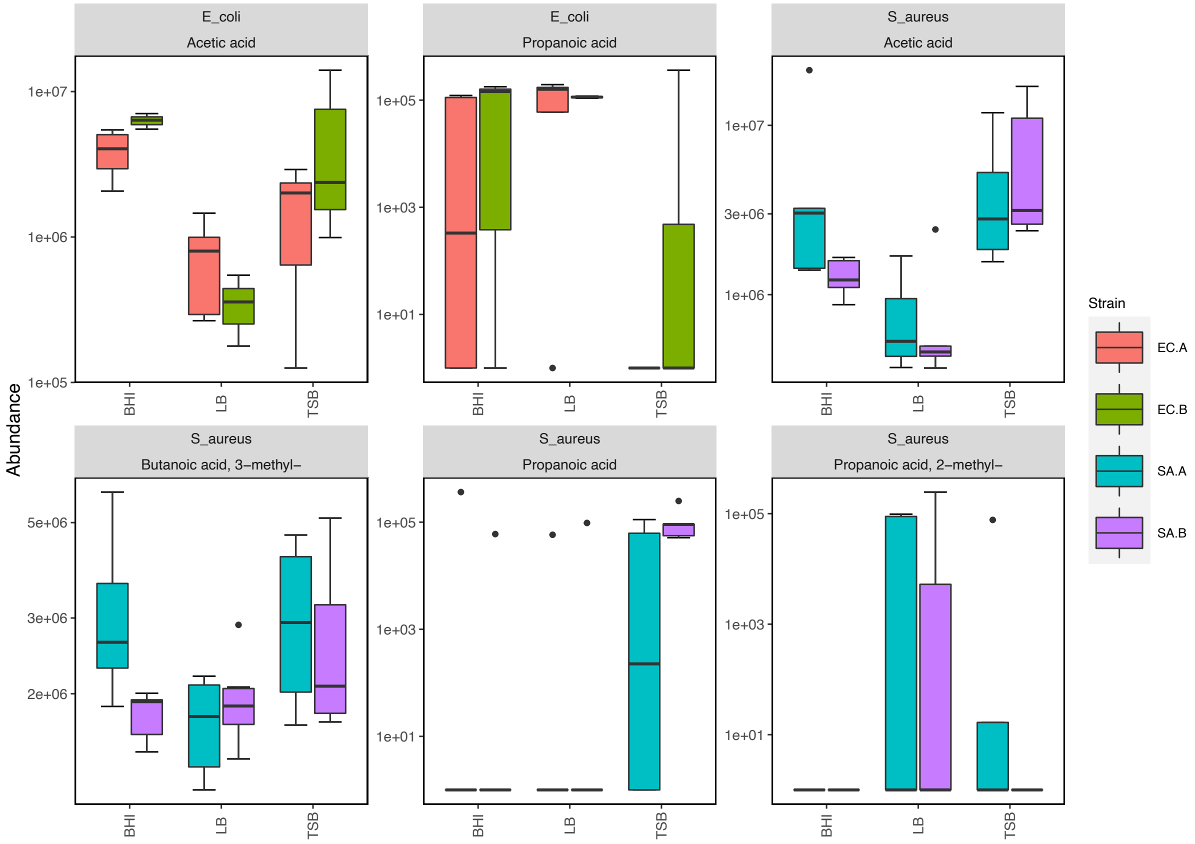

Figure S9 : Species- and acid-specific boxplots. Each plot illustrates the abundance of an individual acidic compound emitted by two strains of a species (*E. coli* and *S. aureus*) across three nutrient-rich media (Brain Heart Infusion – BHI; Lysogeny Broth – LB; Tryptone Soy Broth – TSB). Each strain is colour-coded according to the legend at the left side of the plot. *For each examined strain in BHI, n=5;, TSB, n=5; and LB media, n = 5. *Five replicates were analysed for each strain in each media except for E. coli (EC.A and EC.B) in BHI (n = 4) and P. aeruginosa (PA.B) in TSB (n = 3).*

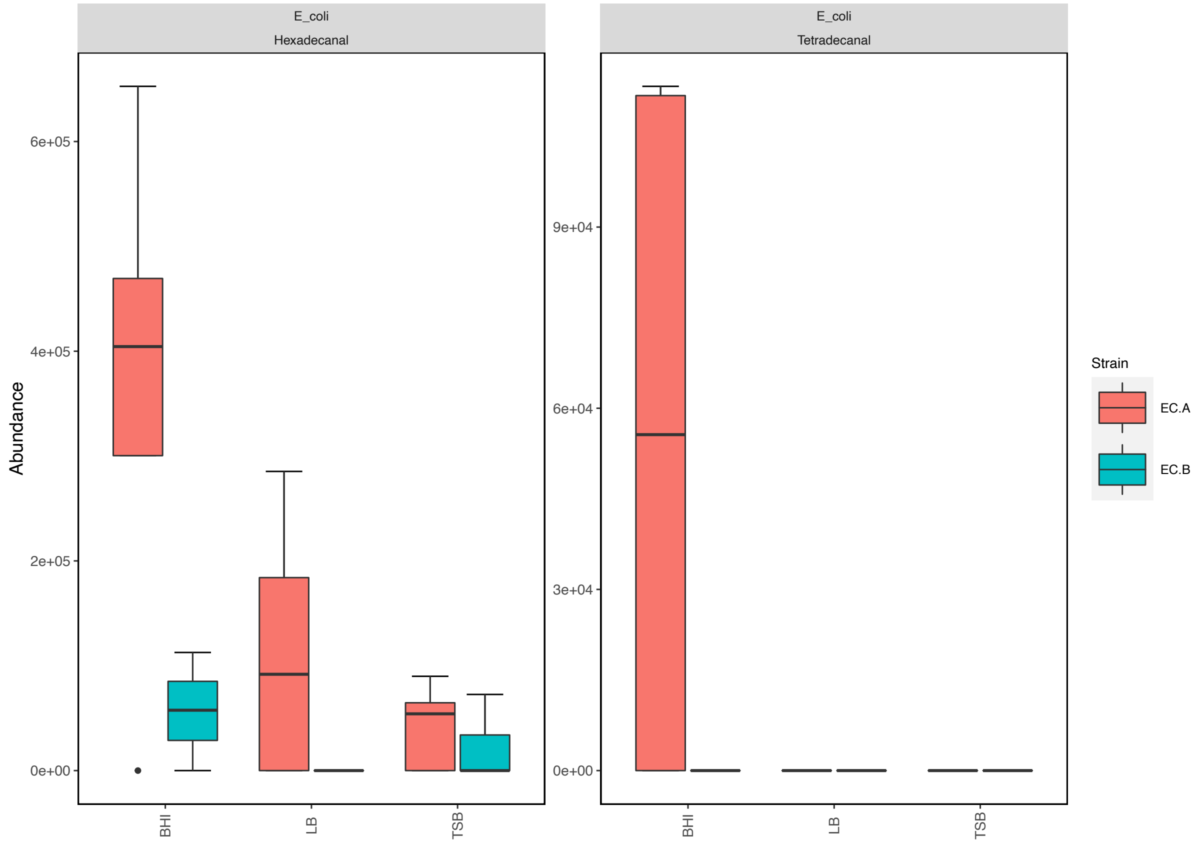

Figure S10 : Species- and aldehyde-specific boxplots. Each plot illustrates the abundance of an individual aldehydic compound emitted by two strains of a species (*E. coli, P. aeruginosa*, and *S. aureus*) across three nutrient-rich media (Brain Heart Infusion – BHI; Lysogeny Broth – LB; Tryptone Soy Broth – TSB). Each strain is colour-coded according to the legend at the left side of the plot. *For each examined strain in BHI, n=5;, TSB, n=5; and LB media, n = 5. *Five replicates were analysed for each strain in each media except for E. coli (EC.A and EC.B) in BHI (n = 4) and P. aeruginosa (PA.B) in TSB (n = 3).*

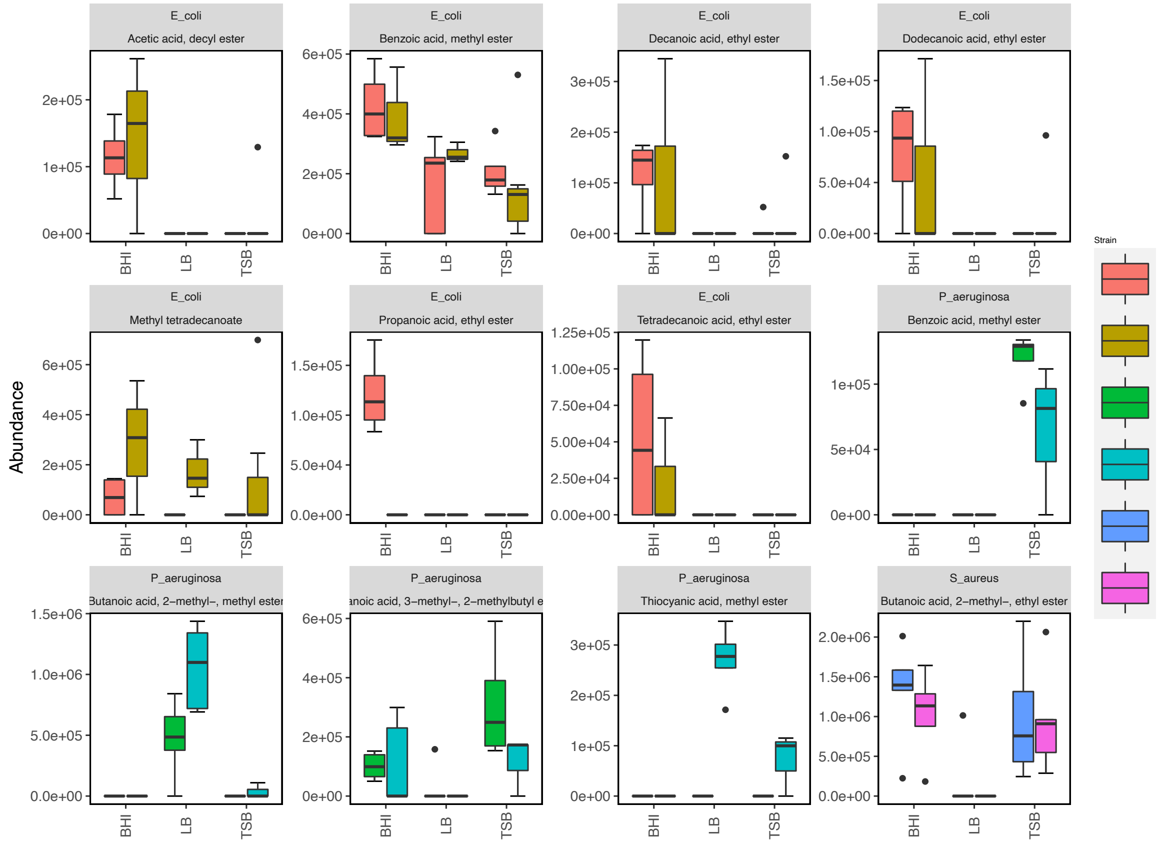

Figure S11 : Species- and ester-specific boxplots. Each plot illustrates the abundance of an individual fatty acid ester compound emitted by two strains of a species (*E. coli, P. aeruginosa*, and *S. aureus*) across three nutrient-rich media (Brain Heart Infusion – BHI; Lysogeny Broth – LB; Tryptone Soy Broth – TSB). Each strain is colour-coded according to the legend at the left side of the plot. *For each examined strain in BHI, n=5;, TSB, n=5; and LB media, n = 5. *Five replicates were analysed for each strain in each media except for E. coli (EC.A and EC.B) in BHI (n = 4) and P. aeruginosa (PA.B) in TSB (n = 3). *The name of the second compound in the third row is 'Butanoic acid, 3-methyl-, 2-methylbutyl ester'.*

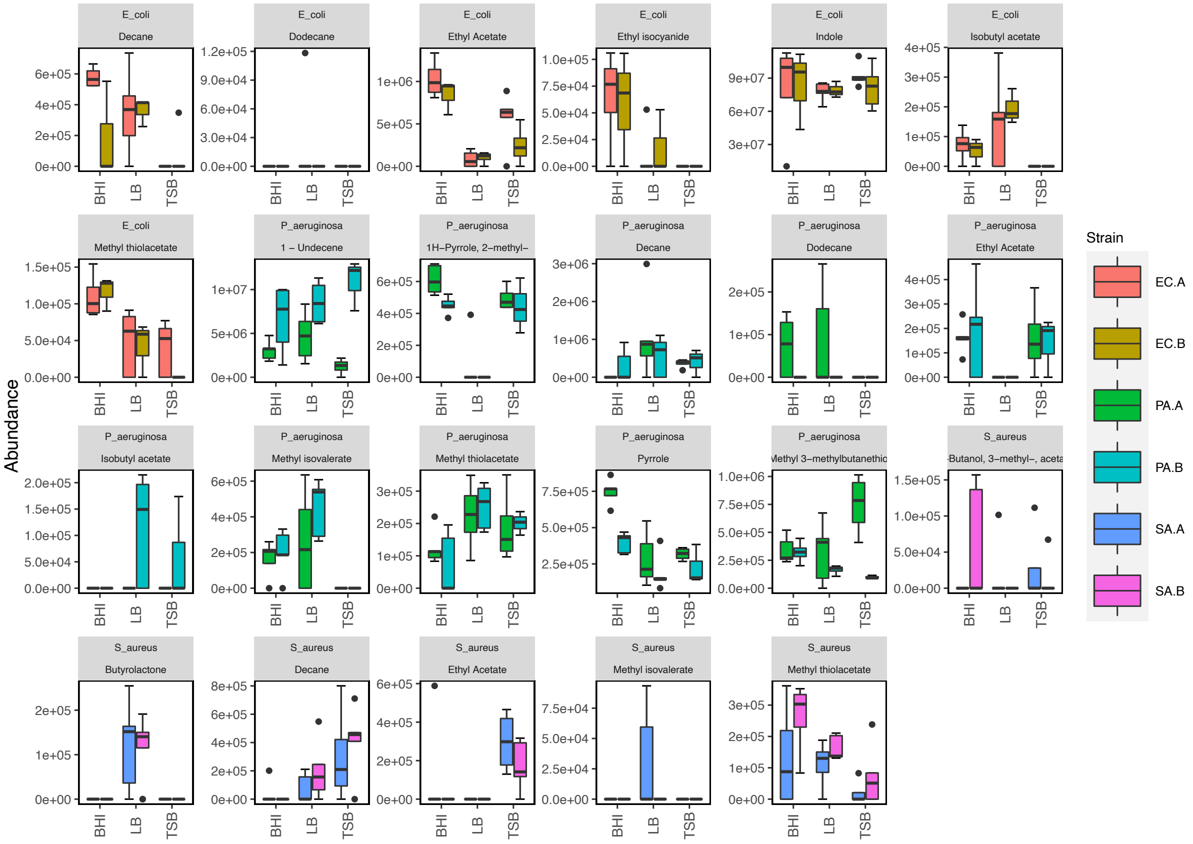

Figure S12 : Species- and compound-specific boxplots. Each plot illustrates the abundance of an individual compound emitted by two strains of a species (*E. coli, P. aeruginosa*, and *S. aureus*) across three nutrient-rich media (Brain Heart Infusion – BHI; Lysogeny Broth – LB; Tryptone Soy Broth – TSB). Each strain is colour-coded according to the legend at the left side of the plot. Compounds shown in this plot belong to a variety of chemical classes. *For each examined strain in BHI, n=5;, TSB, n=5; and LB media, n = 5. *Five replicates were analysed for each strain in each media except for E. coli (EC.A and EC.B) in BHI (n = 4) and P. aeruginosa (PA.B) in TSB (n = 3).*

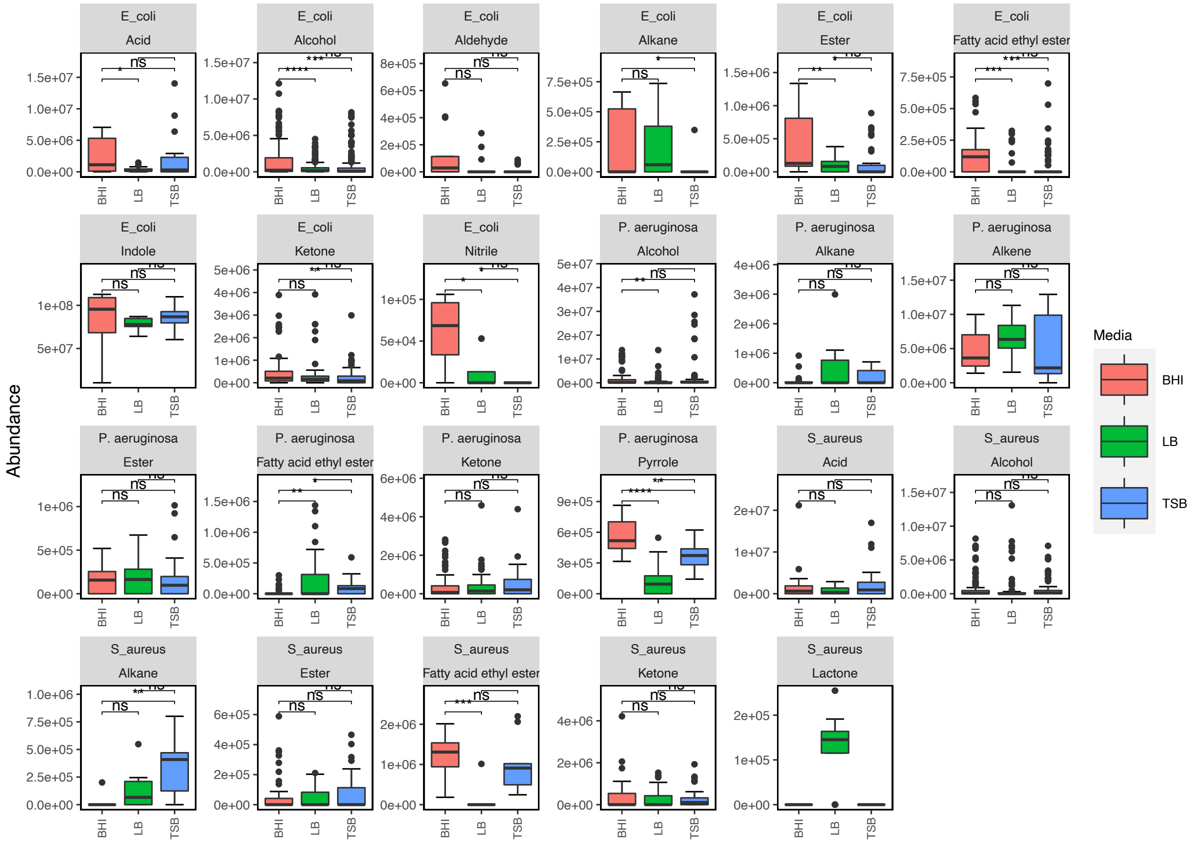

Figure S13 : Grouped boxplot representation *illustrating the differences in emission of individual chemical classes in BHI, LB and TSB growth media by E. coli, P. aeruginosa and S. aureus. This bar plot was obtained by summing the mean abundance of each chemical class detected in each of the examined bacteria. The following symbols were used to indicate statistical significance (*ns: p > 0.05; *: p <= 0.05; **: p <= 0.01; ***: p <= 0.001; ****: p <= 0.0001). In row 1, compound 1, '*' between LB and TSB is not visible. In row 3, compound 2, '*' between LB and TSB is not visible. In row 5, compound 3, '**' between LB and TSB is not visible.

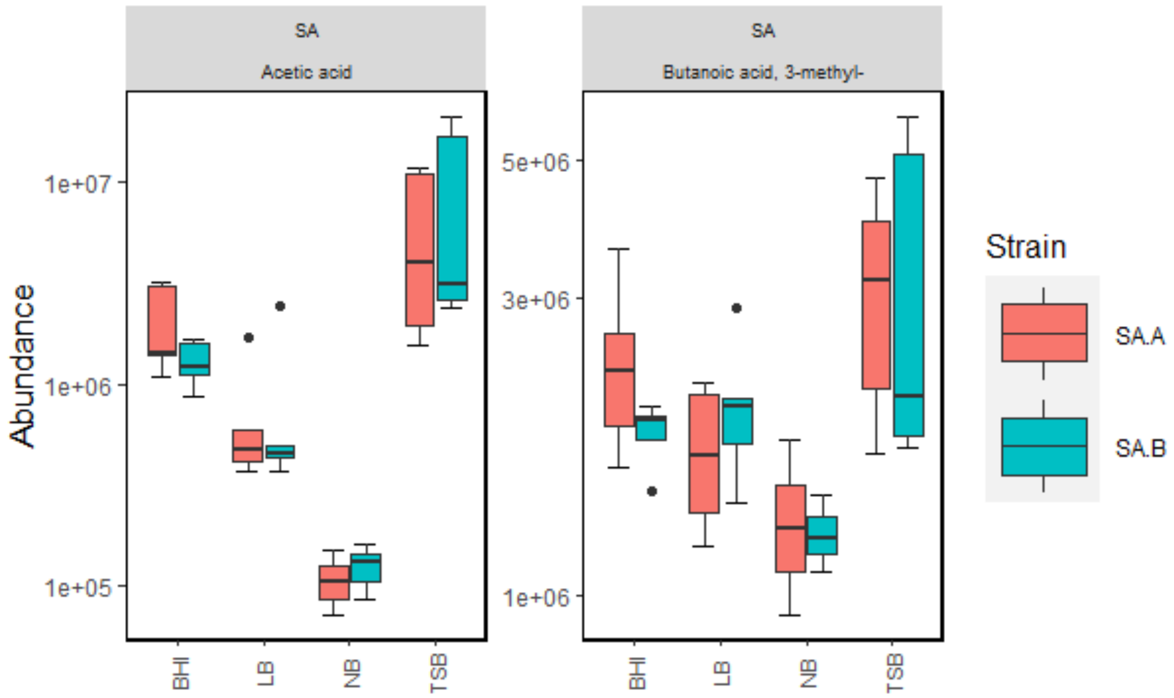

Figure S14 : S. aureus - specific box plots illustrating the difference in acid abundances between glucose-containing media (BHI and TSB) and glucose-free media (LB and NB). Nutrient broth – NB

Table S1: List of contaminant peaks present in background media, fiber and column controls. 'X' marks presence of compound in chromatograms

Compound	Base peak	BHI	LB	TSB	Fiber blank
Acetone	43	X	X	X	
Cyclotrisiloxane, hexamethyl-	207	-	-	-	X
Cyclotetrasiloxane, octamethyl	281	-	-	-	X
Furan, 3-methyl-	82	X	-	X	-
2,4-Dimethyl-1-heptene	70	-	-	X	-
2-Butanone	43	X	X	X	-
Butanal, 2-methyl-	57	X	X	X	-
Butanal, 3-methyl-	41	X	X	X	-
Benzene	78	X	-	-	-
2,3-Butanedione	43	X	X	X	-
Methyl Isobutyl Ketone	43	X	-	-	-
2-Butanol	45	X	-	X	-
Trichloromethane	83	X	-	-	-
Toluene	91	X	X	X	-
Disulfide, dimethyl	94	X	X	X	-
2-Butenal, 2-methyl-	84	X	X	X	-
1-Butanol	56	X	X	X	-
Pyrazine	80	X	-	X	-

Styrene	104	X	-	X	-
Pyrazine, methyl-	94	X	-	X	-
2-Propanone, 1-hydroxy-	43	X	-	X	-
Pyrazine, ethyl-	107	X	-	X	-
Pyrazine, 2,5-dimethyl	108	X	X	X	-
Pyrazine, 2,6-dimethyl-	108	-	-	X	-
Dimethyl trisulfide	126	X	X	X	-
Pyrazine, 2-ethyl-5-methyl-	121	X	X	X	-
Pyrazine, trimethyl-	122	X	-	X	-
Pyrazine, 3-ethyl-2,5-dimethyl-	135	X	-	X	-
Acetic acid	43	-	-	X	-
3-Furaldehyde	95	X	-	-	-
Nonanal	57	X	X	X	X
1-Hexanol, 2-ethyl-	57	X	X	X	X
Decanal	57	X	X	X	X
Benzaldehyde	106	X	X	X	X
Silanediol, dimethyl-	77	X	X	X	X
Oxime-, methoxy-phenyl-_	133	X	X	X	X
2-Furanmethanol	98	X	-	X	-
Benzaldehyde, 4-methyl-	119	X	-	-	-
2-Acetylthiazole	43	X	-	-	-
Furan, 3-phenyl	115	-	-	-	-

Table S2: Agilent MassHunter parameters used for chromatographic data analysis

Chromatographic parameters	
Peak finding method	*Chromatogram deconvolution*
Peak filters	*>= 50000 counts*
Peak area calculation	*Chromatogram deconvolution*
Compound identification	*NIST Mass Spectral Library 2017*
Signal smoothing	*No signal smoothing was performed*
Baseline correction	*No baseline correction was performed*
